# Characterization of the Metabolic Profile of Olive Tissues (Roots, Stems and Leaves): Relationship with Cultivars’ Resistance/Susceptibility to the Soil Fungus *Verticillium dahliae*

**DOI:** 10.3390/antiox12122120

**Published:** 2023-12-15

**Authors:** Irene Serrano-García, Lucía Olmo-García, Olga Monago-Maraña, Iván Muñoz Cabello de Alba, Lorenzo León, Raúl de la Rosa, Alicia Serrano, Ana María Gómez-Caravaca, Alegría Carrasco-Pancorbo

**Affiliations:** 1 Department of Analytical Chemistry, Faculty of Sciences, University of Granada, Ave. Fuentenueva s/n, E-18071 Granada, Spain; iserrano@ugr.es (I.S.-G.); ivanfenty@correo.ugr.es (I.M.C.d.A.); anagomez@ugr.es (A.M.G.-C.); alegriac@ugr.es (A.C.-P.); 2 Department of Analytical Sciences, Faculty of Sciences, Universidad Nacional de Educación a Distancia (UNED), Avda. Esparta s/n, Crta. de Las Rozas-Madrid, E-28232 Madrid, Spain; olgamonago@ccia.uned.es; 3 Instituto de Investigación y Formación Agraria y Pesquera (IFAPA), Centro Alameda del Obispo, Ave. Menéndez Pidal s/n, E-14004 Córdoba, Spain; lorenzo.leon@juntadeandalucia.es (L.L.); or raul.rosa@ias.csic.es (R.d.l.R.); 4 Department of Experimental Biology, The University Institute of Research on Olive and Olive Oils (INUO), University of Jaén, Campus Las Lagunillas s/n, E-23071 Jaén, Spain; segoalicia@gmail.com

**Keywords:** *Olea europaea* L., verticillium wilt, plant metabolomics, LC-MS profiling, secondary metabolites, phenolic compounds, triterpenic compounds

## Abstract

Verticillium wilt of olive (VWO) is one of the most widespread and devastating olive diseases in the world. Harnessing host resistance to the causative agent is considered one of the most important measures within an integrated control strategy of the disease. Aiming to understand the mechanisms underlying olive resistance to VWO, the metabolic profiles of olive leaves, stems and roots from 10 different cultivars with varying levels of susceptibility to this disease were investigated by liquid chromatography coupled to mass spectrometry (LC-MS). The distribution of 56 metabolites among the three olive tissues was quantitatively assessed and the possible relationship between the tissues’ metabolic profiles and resistance to VWO was evaluated by applying unsupervised and supervised multivariate analysis. Principal component analysis (PCA) was used to explore the data, and separate clustering of highly resistant and extremely susceptible cultivars was observed. Moreover, partial least squares discriminant analysis (PLS-DA) models were built to differentiate samples of highly resistant, intermediate susceptible/resistant, and extremely susceptible cultivars. Root models showed the lowest classification capability, but metabolites from leaf and stem were able to satisfactorily discriminate samples according to the level of susceptibility. Some typical compositional patterns of highly resistant and extremely susceptible cultivars were described, and some potential resistance/susceptibility metabolic markers were pointed out.

## 1. Introduction

*Olea europaea* L. is one of the oldest trees that mankind has cultivated. Its derived products, mainly table olives and oil, are consumed practically all over the world, mainly due to its important nutraceutical properties [1]. As a result, olive trees have gained great relevance worldwide, and are considered a very valuable crop providing important economic and ecological benefits [2,3]. However, they are susceptible to various abiotic and biotic stresses. Among them, verticillium wilt of olive (VWO), a disease caused by the soil-born fungus *Verticillium dahliae*, currently represents the main phytosanitary limitation in many olive-growing areas. This fungus enters the olive tree through the roots and spreads to the trunk, branches and leaves, where it blocks the flow of water and nutrients through the xylem vessels, causing plant wilting and, eventually, the death of the infected tree [4]. Thus, VWO can severely affect the growth and yield of olives, leading to significant economic losses [5]. *V. dahliae* can persist in soil for years, making it difficult to control once established [6]. In the absence of effective preventive or curative chemical fungicides, integrated approaches involving the use of multiple before and after planting practices need to be implemented to reduce the incidence of the disease [6,7,8]. One of the most important practices is the use of resistant cultivars. Although a wide genetic variability has been reported in the olive germplasm [9], very few cultivars have shown a high level of resistance to VWO [10]. Therefore, several breeding efforts have been developed to produce new cultivars with a high level of resistance to VWO combined with good agronomic characteristics [10,11,12,13]. 

To improve the efficiency of those breeding programs, it would be of interest to uncover the genetic and metabolic pathways involved in VWO resistance [6,14]. In this sense, all plants develop a defence strategy upon pathogen attack, which triggers a set of multi-component responses, including the production of signalling molecules such as reactive oxygen species, induction of the antioxidant system, activation of pathways that generate anti-fungal secondary metabolites and others [15]. Moreover, a higher resistance of some olive cultivars to VWO infection has been correlated with enhanced enzymatic activities related to cell-wall reinforcement and the up-regulation of plant hormones involved in the induction of innate systemic resistance [16]. The functional traits of olive roots (biomass allocation, dry matter content and root system architecture) have been also associated with the resistance level to VWO [17], and a differential basal set of genes and diverse transcriptomic responses have been found in roots of resistant and susceptible cultivars [14,18]. Therefore, the evidence suggests that there are distinct biochemical and physiological differences between susceptible and resistant cultivars. These differences exist at the genome, transcriptome and metabolome levels, and can be constitutive (basal) or induced by the fungus–plant interaction [16].

Secondary metabolites are not directly involved in the growth and development of plants, but are synthesized for specific ecological roles, such as defence against pathogens or abiotic constraints [19,20]. The exact mechanisms by which secondary metabolites confer resistance to *V. dahliae* are not yet fully understood. However, it has been proposed that these compounds may act by inhibiting the growth and development of the fungus, by modulating the plant’s immune response, or by enhancing the plant’s tolerance to stress [19,21]. Some olive secondary metabolites, such as rutin, oleuropein, luteolin-7-glucoside and hydroxytyrosol have shown in vitro antifungal activity against *V. dahliae* [22,23].

The accumulation of phenolic compounds in different olive organs after *V. dahliae* inoculation has been repeatedly reported [21,22,24,25]. Even though most of the cited reports focused on the total phenolic and total o-diphenols content (determined by colorimetric methods), some information has also been gathered about the role of specific compounds on olive defence against this soil-borne fungus. For example, Báidez and collaborators found that infected stem tissues presented higher levels of oleuropein, rutin and luteolin 7-glucoside than the tissues from healthy plants [22]. Contrastingly, Markakis and co-workers found a negative correlation among oleuropein content and relative fungus DNA quantity in infected roots. On the contrary, the same authors described an increase in the verbascoside concentration in roots after *V. dahliae* infection, showing an opposite behaviour of the two major root metabolites [26]. Recently, Cardoni and co-authors found only minor significant changes in the metabolic profile of roots after *V. dahliae* inoculation [27]. 

In addition to the induced changes in secondary metabolites, the study of the basal composition of olive tissues is relevant to understanding the mechanisms underlying resistance/susceptibility to VWO [27]. A positive association has been found between total basal polyphenol content in olive leaves, stems and roots and the resistance level of the cultivar [24,25]. However, little information is available on the correlation between the basal content of specific metabolites and the cultivar’s resistance to *V. dahliae* infection. A negative association between the root content of verbascoside, maslinic acid and methoxypinoresinol glucoside and the tolerance level to VWO of ‘Picual’, ‘Hojiblanca’ and ‘Lechín de Sevilla’ cultivars has been recently reported [27]. The same authors also described higher concentrations of oleuropein, oleuropein aglycone, ligstroside and elenolic acid glucoside in roots of VWO-tolerant varieties (‘Changlot Real’, ‘Empeltre’ and ‘Frantoio’). Nonetheless, a thorough metabolomic analysis of various plant tissues in cultivars exhibiting a broad range of responses to VWO has yet to be conducted. 

Metabolomics represents a powerful tool to explore the metabolic profiles of cultivars showing varying levels of resistance/susceptibility to VWO and to assess the distribution of secondary metabolites among different olive tissues involved in *V. dahliae* infection and spread through the plant. These kinds of analytical approaches have also been proven to be effective in achieving the phenolic characterization of olive leaves of cultivars resistant to Xylella, both at a baseline level and after inoculation [28,29]. Thus, the main goals of this work were: (i) to carry out the metabolic profiling of leaves, stems, and roots from 10 olive cultivars showing varying levels of resistance to VWO; (ii) to establish the metabolite distribution in the different tissues affected by the fungus infection; and (iii) to find possible metabolite correlations with resistance/susceptibility to *V. dahliae*.

## 2. Materials and Methods

### 2.1. Chemicals and Standards

Deionized water (resistivity 18.2 MΩ cm) was produced with a Millipore Milli-Q system (Bedford, MA, USA). Gradient grade ethanol and LC-MS grade acetonitrile were supplied by Prolabo (Paris, France). Acetic acid and pure standards of olive secondary metabolites (quinic, maslinic, betulinic and oleanolic acids, hydroxytyrosol, tyrosol, luteolin 7-*O*-glucoside, rutin, verbascoside and oleuropein) were purchased from Sigma-Aldrich (St. Louis, MO, USA). 

### 2.2. Plant Material and Samples Pretreatment

One year old plants from 10 different cultivars (‘Arbequina’, ‘Empeltre’, ‘Frantoio’, ‘Hojiblanca’, ‘Jabali’, ‘Koroneiki’, ‘Leccino’, ‘Mastoidis’, ‘Menya’ and ‘Picual’) were obtained by vegetative propagation of semi-hardwood stem cuttings from the World Olive Germplasm Bank of the Centro IFAPA ‘Alameda del Obispo’ in Cordoba, Spain [9]. These cultivars were selected as having different levels of resistance/susceptibility to VWO [6,10], from highly resistant to extremely susceptible: ‘Frantoio’ and ‘Empeltre’, highly resistant (HR); ‘Koroneiki and ‘Leccino’, resistant (R); ‘Arbequina’ and ‘Picual’, moderately susceptible (MS); ‘Hojiblanca’ and ‘Menya’, susceptible (S); ‘Jabali’ and ‘Mastoidis’, extremely susceptible (ES). Roots, stems and leaves were sampled from three plants (biological replicates) from each olive cultivar to get a total number of 90 samples. Plant tissues were washed with water and dried at room temperature in the dark until constant weight. Afterwards, all the samples were ground, sieved through a 0.5 mm metal sieve to obtain a standard particle size and stored at −20 °C. 

### 2.3. Secondary Metabolites Extraction and LC-MS Analysis

The extraction of the fraction of olive secondary metabolites was carried out by applying a previously reported ultrasound-assisted solid–liquid extraction protocol [30] with slight modifications. First, 100 mg of tissue powder was subjected to two consecutive extraction steps with ethanol–water mixtures (60:40 for the first step and 80:20 for the second one) followed by a third step with pure ethanol. Leaf samples required a volume of 10 mL of the extractant agent in each extraction step, whereas roots and stems were extracted with 5 mL of solvent per cycle. Each extraction cycle involved 30 min of ultrasound extraction, centrifugation at 8603× *g* for 10 min and upper phase separation. Finally, 1 mL aliquots of the combined supernatants were filtered with 0.22 μm Clarinert^®^ nylon syringe filters (Agela Technologies, Torrace, CA, USA) and transferred to amber glass HPLC vials.

External calibration curves (0.1–500 mg L^−1^) of commercially available standards were prepared in ethanol–water (80:20) and used for the quantification of the analytes of interest. Standard solutions and quality controls (QC) of each matrix, which were prepared by mixing a portion of solid powder from all the samples included in the study (per tissue type), were used to assess the main analytical parameters of the method as well as to assess the performance of the analytical system during the analysis sequence. All the stock solutions and plant extracts were stored at −20 °C until analysis.

LC-MS analyses were conducted on two different systems. First, the qualitative characterization of samples’ metabolic profiles was carried out on a Waters Acquity UPLC H–Class system coupled to a QTOF SYNAPT G2 mass spectrometer (Waters, Manchester, UK). Second, an Agilent 1260 LC system (Agilent Technologies, Waldbronn, Germany) coupled to a Bruker Daltonics Esquire 2000 IT mass spectrometer (Bruker Daltonik, Bremen, Germany) was used for quantitative purposes.

Chromatographic and MS detection conditions were adapted from those presented in a previous report [31]. Regardless of the employed LC-MS system, metabolite separation was carried out on a Zorbax Extend C18 column (100 × 4.6 mm, 1.8 μm particle size, Agilent Technologies) operated at 40 °C, with a sample injection volume of 10 μL. A mobile phase gradient of water (Phase A) and acetonitrile (Phase B)—both acidified with 1% acetic acid—was applied for the elution of compounds at a flow rate of 1 mL min^−1^: 0–10 min, 10–25% B; 10–12 min, 25–60% B; 12–14 min, 60–80% B; 14–18 min, 80–100% B (kept for 2 min), 20–21 min, 100–10% B (kept for 3 min of equilibration time). The LC flow was diverted (1:4) to the electrospray interface, and source parameters were accordingly selected, depending on the MS instrument used as analyser: +3.2 kV of capillary voltage, 30 psi of nebulizer pressure, 300 °C and 9 L min^−1^ of drying gas temperature and gas flow, respectively, on the ESI-IT-MS spectrometer, and +2.1 kV of capillary voltage, 100 °C of source temperature, 50 L h^−1^ of cone gas flow, 500 °C and 1000 L h^−1^ of desolvation temperature and gas flow, apiece, on the ESI-QTOF-MS platform. Full scan spectra (50–1200 Da) were recorded in negative polarity with both detectors.

### 2.4. Data Treatment

Instrument control and chromatographic data treatment were carried out with the software ChemStation B.04.03 (Agilent Technologies, Waldbronn, Germany), Esquire Control and Data Analysis 4.0 (Bruker Daltonik, Bremen, Germany), and MassLynx 4.4 (Waters). Quantitative data were expressed as mean ± standard deviation (*n* = 3) in mg kg^−1^ of dry weight (DW). Analysis of variance (one-way ANOVA) was conducted using the statistical software InfoStat 2020. Statistical significance was defined as *p*-values less than α = 0.05 using the Tukey’s post hoc test. Graphical representations were performed with the software Excel 2021 (v.18.0). In order to explore the variation in data between varieties with different resistance to VWO, PCA was applied using The Unscrambler, version 6.11 (CAMO Software AS, Oslo, Norway). After that, to evaluate the possibility of discriminating samples according to the level of VWO resistance, PLS-DA was employed as a classification algorithm [32] using Matlab R2007b (The MathWorks, Inc., Natick, MA, USA) with the PLS_Toolbox 5.51 (Eigenvector Research Inc. Wenatchee, WA, USA). In both cases, data were scaled before the analysis.

## 3. Results and Discussion

### 3.1. Qualitative Characterization of Plant Tissue Metabolic Profiles

In a first stage of this work, the metabolic profiles of roots, stems and leaves from the sampled olive cultivars were comprehensively characterised. The powerful multi-class LC-MS method applied to the analysis of the prepared extracts allowed the monitoring of diverse chemical families in a single run (organic acids, pentacyclic triterpenes and phenolic compounds) [31]. The accurate *m*/*z* and isotopic distribution obtained with the QTOF MS analyser enabled the prediction of the molecular formula of the compounds. Metabolite identification was carried out by comparison with commercial standards (when available), as well as with an in-house built database of *Olea europaea* L. secondary metabolites and existing literature (see Table 1), considering high-resolution MS (HRMS) data, retention time (Rt) and elution order of the detected peaks.

The list of compounds found in the investigated samples is presented in Table 1. It includes the detected *m*/*z* of the [M-H]^−^ ion, the mass error (difference between detected and theoretically calculated *m*/*z* signals), the iFIT value (which gives an idea of the concordance between the experimental and theoretical isotopic patterns), the calculated molecular formula and the identity assigned to each detected peak. Some reports in which the compounds were previously described are cited under the reference heading, and the kind of plant tissue where the compound was quantified in a subsequent step of the project is marked in the appropriate column. It is worth noting that Table 1 lists a good number of examples of very meritorious works in the field, but by no means does it pretend to be a comprehensive review by citing all the references reporting each compound. More than 50 compounds belonging to several chemical classes were, at least, tentatively annotated: 1 organic acid (quinic acid), 3 pentacyclic triterpenes (maslinic, betulinic and oleanolic acids) and 47 phenolic compounds. The last class of metabolites, one of the most ubiquitously distributed in the plant kingdom, gathered the largest number of compounds, which can be classified into 5 subfamilies: 7 lignans (cycloolivil, and two isomers of its glycosidic form, acetoxypinoresinol, acetoxypinoresinol glucoside, hydroxypinoresinol glucoside and methoxypinoresinol glucoside), 16 flavonoids (dihydroquercetin 3-*O*-glucoside, gallocatechin, cyanidin *O*-glucoside, rutin, taxifolin, three isomers of quercetin *O*-glucoside, three isomers of luteolin *O*-glucoside, apigenin *O*-rutinoside, apigenin 7-*O*-glucoside, diosmin, dihydrokaempferol and chrysoeriol *O*-glucoside), 5 simple phenols and glucoside derivatives (hydroxytyrosol and its glycosidic form, verbascoside, isoverbascoside and phenylethyl *β*-primeveroside (which has been included in this subfamily because of its structural similarity), 2 iridoids (7-deoxyloganic acid and 11-hydroxyiridodial glucoside pentaacetate) and 17 secoiridoids and related compounds. The group of secoiridoids was the most abundant subfamily of phenolic compounds and comprised lucidumoside C, ligstroside, 4 isomers of a compound with molecular formula C_31_H_42_O_18_, which could be either neonuzhenide or oleuropein glucoside, several oleuropein related compounds (oleuropein, demethyl oleuropein, hydroxy oleuropein, two isomers of oleuropein aglycone, and oleuroside) and 5 derivatives of elenolic acid (two isomers of elenolic acid glucoside, the aldehydic form of decarboxymethyl elenolic acid glucoside, oleoside and secologanoside). The latter two are mass isomers whose identity was assigned based on the relative retention times described in previous reports [33,34]. As seen in Table 1, a large proportion of the identified compounds were glycosylated derivatives and isomers with hexoses attached in different positions that could not be determined on the basis of the HRMS data exclusively, when the pure standards were not available.

Some other compounds could not be confidently annotated but are presented in Table 1 because of their relevance within the profiles (in terms of peak area). The compound with *m*/*z* 625.1977 eluting at 2.4 min (unknown 1) presented C_25_H_38_O_18_ as the calculated molecular formula. Interestingly, it produced two major in-source fragments, corresponding to C_17_H_24_O_11_ (elenolic acid glucoside) and C_23_H_34_O_16_ (elenolic acid diglucoside), which suggest that it might be an elenolic acid diglucoside derivative (+C_2_O_2_H_4_). The molecular formula calculated for unknown 3 (Rt: 9.2 min and *m*/*z* 491.1769) was C_21_H_32_O_13_. Such metabolite has not been described before in any olive matrix to the best of our knowledge; nevertheless, it could correspond to a phenolic glycoside, such as 3,4,5-trimethoxyphenyl 2-*O-*(*α*-L-fucopyranosyl)-*β*-D-glucopyranoside from *Walsura yunnanensis* [35]. In the same way, unknown 4 (Rt: 11.5 min and *m*/*z* 651.2283), with a calculated molecular formula of C_31_H_40_O_15_ could be annotated as martynoside, a verbascoside derivative previously isolated from *Buddleja globosa* hope [36]. Regarding unknown 5 (Rt: 16.4 min and *m*/*z* 617.3840), with a calculated molecular formula of C_39_H_54_O_6_, as it eluted in the chromatogram area of triterpenic acids, it may be some kind of derivative, such as the ester caffeoyl-oleanolic acid that was isolated from *Dioclea lasiophylla* by David and coauthors [37]. Another unidentified compound (unknown 2), presenting a major signal with *m*/*z* 511.3484 (calculated molecular formula: C_25_H_52_O_10_), eluted at 6.7 min, but no plausible identity could be suggested for this metabolite.

**Table 1:** List of metabolites detected in root, stem and leaf extracts by LC-ESI-QTOF MS profiling.

Rt/min	Experimental *m*/z *	Error/mDa	iFIT	Molecular Formula	Name of the Compound	Chemical Family	References	Quantified in:		
								Leaf	Stem	Root
0.8	191.0557	0.1	418.2	C_7_H_12_O_6_	quinic acid	organic acids	standard	x	x	x
1.0	389.1083	−0.1	266.9	C_16_H_22_O_11_	oleoside	secoiridoids and derivatives	[33,34]	x	x	x
1.3	315.1078	−0.2	373.2	C_14_H_20_O_8_	hydroxytyrosol glucoside	simple phenols and derivatives	[28,30,38,39]	x	x	
1.4	153.0551	−0.1	260.2	C_8_H_10_O_4_	hydroxytyrosol	simple phenols and derivatives	standard	x		
1.8	465.1035	0.2	277.6	C_21_H_22_O_12_	dihydroquercetin 3-*O*-glucoside	flavonoids	[28]		x	
2.3	389.1082	−0.2	527.9	C_16_H_22_O_11_	secologanoside	secoiridoids and derivatives	[28,33,34]	x	x	x
2.4	625.1977	−0.3	376.0	C_25_H_38_O_18_	unknown 1	unknown	-	x	x	x
3.0	305.0670	−0.28	313.2	C_15_H_14_O_7_	gallocatechin	flavonoids	[30,40,41]	x		
3.0	449.1086	0.2	122.6	C_21_H_22_O_11_	cyanidin *O*-glucoside	flavonoids	[30,42]		x	
3.5	403.1236	−0.4	479.2	C_17_H_24_O_11_	elenolic acid glucoside (isomer 1)	secoiridoids and derivatives	[28,33,39,43]	x	x	x
3.9	377.1447	−0.1	337.0	C_16_H_26_O_10_	aldehydic form of decarboxymethyl elenolic acid glucoside	secoiridoids and derivatives	[44]	x	x	
3.9	537.1974	0.2	275.9	C_26_H_34_O_12_	cycloolivil glucoside (isomer 1)	lignans	[41,45,46]			x
4.7	403.1239	−0.1	458.3	C_17_H_24_O_11_	elenolic acid glucoside (isomer 2)	secoiridoids and derivatives	[28,33,39,43]	x	x	x
4.8	537.1976	0.4	125.2	C_26_H_34_O_12_	cycloolivil glucoside (isomer 2)	lignans	[46]			x
5.1	415.1607	0.3	529.8	C_19_H_28_O_10_	phenylethyl primeveroside	simple phenols and derivatives	[30]		x	
5.6	525.1604	−0.4	639.1	C_24_H_30_O_13_	demethyl oleuropein	secoiridoids and derivatives	[33,41,44]	x	x	
5.7	609.1453	−0.3	427.3	C_27_H_30_O_16_	rutin	flavonoids	standard	x	x	
5.8	359.1341	−0.1	513.8	C_16_H_24_O_9_	7-deoxyloganic acid	iridoid	[40,41,46]			x
6.1	555.1711	−0.3	302.3	C_25_H_32_O_14_	hydroxy oleuropein	secoiridoids and derivatives	[28,30,39,41]		x	x
6.2	303.0506	0.1	166.6	C_15_H_12_O_7_	taxifolin	flavonoids	[30,38,47,48]		x	
6.2	463.0874	−0.3	393.1	C_21_H_20_O_12_	quercetin *O*-glucoside (isomer 1)	flavonoids	[30,41,48]		x	
6.3	375.1444	0.0	113.0	C_20_H_24_O_7_	cycloolivil	lignans	[42,45,46]		x	
6.4	701.2291	−0.2	570.0	C_31_H_42_O_18_	neonuzhenide/oleuropein glucoside (isomer 1)	secoiridoids and derivatives	[28,41]	x	x	x
6.4	447.0923	−0.4	308.2	C_21_H_20_O_11_	luteolin 7-*O*-glucoside (isomer 1)	flavonoids	standard	x	x	
6.7	511.3484	0.2	161.5	C_25_H_52_O_10_	unknown 2	unknown	-	x	x	x
6.8	623.1977	0.1	450.0	C_29_H_36_O_15_	verbascoside	simple phenols and derivatives	standard	x	x	x
7.4	577.1561	0.4	403.9	C_27_H_30_O_14_	apigenin *O*-rutinoside	flavonoids	[41,43]	x		
7.5	623.1975	−0.1	323.9	C_29_H_36_O_15_	isoverbascoside	simple phenols and derivatives	[33,34,41]			x
7.8	447.0925	−0.2	407.7	C_21_H_20_O_11_	luteolin *O*-glucoside (isomer 2)	flavonoids	[39,41]	x	x	
7.9	535.1810	−0.6	257.2	C_26_H_32_O_12_	hydroxypinoresinol glucoside	lignans	[28,41,45,46]		x	x
7.9	701.2290	−0.3	275.2	C_31_H_42_O_18_	neonuzhenide/oleuropein glucoside (isomer 2)	secoiridoids and derivatives	[28,39,41]		x	x
8.0	463.0882	0.5	380.5	C_21_H_20_O_12_	quercetin *O*-glucoside (isomer 2)	flavonoids	[30,34]		x	
8.0	431.0976	−0.2	395.2	C_21_H_20_O_10_	apigenin 7-*O*-glucoside	flavonoids	standard	x		
8.1	565.1923	0.2	283.0	C_27_H_34_O_13_	methoxypinoresinol glucoside	lignans	[45]		x	x
8.2	607.1666	0.3	187.2	C_28_H_32_O_15_	diosmin	flavonoids	[40,41]	x		
8.3	287.0551	−0.5	351.0	C_15_H_12_O_6_	dihydrokaempferol	flavonoids	[30]		x	
8.3	701.2288	−0.5	333.7	C_31_H_42_O_18_	neonuzhenide/oleuropein glucoside (isomer 3)	secoiridoids and derivatives	[28]	x		
8.6	461.1080	−0.4	50.3	C_22_H_22_O_11_	chrysoeriol *O*-glucoside	flavonoids	[30,40,43]	x		
8.7	577.1921	0.0	165.0	C_28_H_34_O_13_	acetoxypinoresinol glucoside	lignans	[41,45]		x	x
8.8	447.0924	−0.3	329.7	C_21_H_20_O_11_	luteolin *O*-glucoside (isomer 3)	flavonoids	[28,30,39]	x	x	
9.0	463.0881	0.4	412.2	C_21_H_20_O_12_	quercetin *O*-glucoside (isomer 3)	flavonoids	[30,34]		x	
9.2	491.1769	0.4	370.4	C_21_H_32_O_13_	unknown 3	unknown	-			x
9.5	701.2296	−0.3	43.1	C_31_H_42_O_18_	neonuzhenide/oleuropein glucoside (isomer 4)	secoiridoids and derivatives	[28]	x	x	x
9.8	539.1762	−0.2	646.3	C_25_H_32_O_13_	oleuropein	secoiridoids and derivatives	standard	x	x	x
10.3	555.2076	−0.2	352.7	C_26_H_36_O_13_	11-hydroxyiridodial glucoside pentaacetate	iridoid	[49]		x	x
10.8	539.1764	−0.1	513.6	C_25_H_32_O_13_	oleuroside	secoiridoids and derivatives	[33,41]	x	x	
11.5	583.2023	−0.4	211.3	C_27_H_36_O_14_	lucidumoside C	secoiridoids and derivatives	[28,30,46]	x	x	x
11.8	523.1817	0.1	60.1	C_25_H_32_O_12_	ligstroside	secoiridoids and derivatives	[28,30,41]	x	x	x
11.5	651.2283	−0.6	374.7	C_31_H_40_O_15_	unknown 4	unknown	-		x	
12.7	415.1392	−0.1	448.8	C_22_H_24_O_8_	acetoxipinoresinol	lignans	[33]		x	x
12.7	377.1235	−0.1	350.2	C_19_H_22_O_8_	oleuropein aglycone (isomer 1)	secoiridoids and derivatives	[30,41]	x	x	
13.3	377.1239	0.3	54.8	C_19_H_22_O_8_	oleuropein aglycone (isomer 2)	secoiridoids and derivatives	[30,41]	x	x	
15.7	471.3467	−0.7	607.4	C_30_H_48_O_4_	maslinic acid	pentacyclic triterpenes	standard	x	x	x
16.4	617.3840	−0.2	101.8	C_39_H_54_O_6_	unknown 5	unknown	-	x	x	
17.5	455.3527	0.2	241.1	C_30_H_48_O_3_	betulinic acid	pentacyclic triterpenes	standard	x	x	x
17.8	455.3526	0.1	282.5	C_30_H_48_O_3_	oleanolic acid	pentacyclic triterpenes	standard	x	x	x

Rt, retention time; * *m*/*z* values correspond to [M−H]^−^.

### 3.2. Quantitative Analysis of the Targeted Metabolites

In a subsequent stage, the prepared extracts were studied from a quantitative point of view, to assess the abundance and distribution of the most relevant metabolites in the three different plant tissues under investigation (leaves, stems and roots) and to seek out differences in the metabolic profiles of the evaluated cultivars. Firstly, serial dilutions of a standard solution containing 10 pure standards of some of the detected analytes were injected into the LC-IT MS system and the main analytical parameters of the quantitative method (linear dynamic range, limits of detection and quantification and repeatability) were assessed to ensure the quality of the obtained results (Table S1). Limits of detection and quantification were found between 2.0–171.3 μg L^−1^ and 6.7–571.0 μg L^−1^ for betulinic acid and tyrosol, respectively. The intra-day repeatability, expressed as coefficient of variation (%CV), presented values between 0.3 and 7.1% for quinic acid and hydroxytyrosol, respectively, and the inter-day repeatability was, in all cases, less than 10.6%, which indicates that the applied methodology exhibited very satisfactory precision.

After evaluating the basic quality parameters of the method, all the prepared extracts were injected into the LC-IT MS system and quantitative data were generated for 56 analytes (28 compounds in roots, 44 in stems and 34 in leaves). The area of compounds lacking an available pure standard was compared to the external calibration curve of a different compound belonging to the same metabolite subfamily or presenting a chemical structure of a similar molecular weight. In this way, luteolin 7-*O*-glucoside was used to quantify all the flavonoids except for rutin and apigenin *O*-rutinoside, which were quantified with the rutin calibration curve; hydroxytyrosol was used to quantify its glycosidic derivative and oleuropein was used to quantify the rest of phenolic compounds and unknowns. Even though no absolute quantification was performed, this strategy enabled the fair comparison of metabolite profusion in the three olive matrices, as well as among the different cultivars.

#### 3.2.1. Metabolites Distribution throughout Plant Tissues

If we focus on the total amount of the quantified compounds, the leaf was the tissue presenting the greatest concentration of the targeted metabolites, the root was the olive organ that had the lowest concentration of this type of compounds and the stem was the matrix with the richest profile in terms of the number of detected molecules. The latter had been observed by Tóth and co-workers, who detected 41 metabolites in olive barks by LC-MS and just 32 of them in leaves [38]. Secoiridoids were the most abundant chemical family and oleuropein was the compound most concentrated in all samples, as widely reported before [38,39,41,47,50,51], except for in ‘Hojiblanca’ and ‘Picual’ roots, where a higher content of verbascoside was found. This finding is in accordance with the results from Cardoni and collaborators [27], and those from Mechri and collaborators, who found a higher concentration of verbascoside in roots of well-watered ‘Chétoui’ olive trees [52].

**Figure 1:**
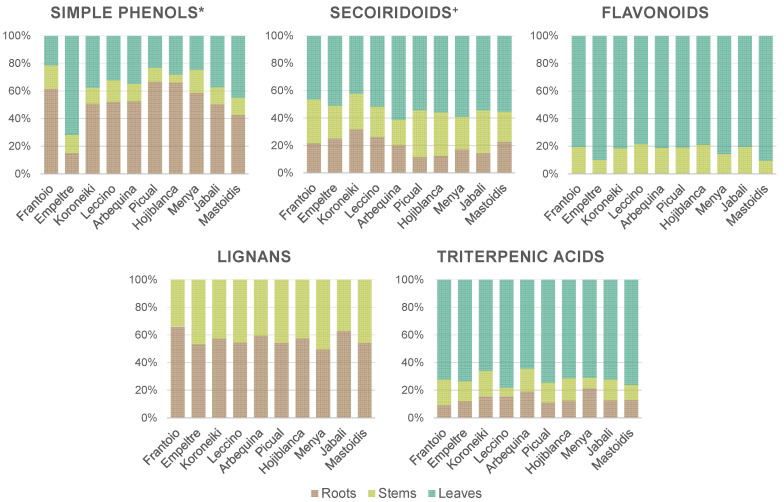
Metabolite distribution (percentage of total amount) throughout the three analysed tissues (roots, stems and leaves) of 10 different olive cultivars (sorted by resistance to VWO: from highly resistant (HR, left) to extremely susceptible (ES, right)). Contents expressed in a normalized way. * and glycoside derivatives ^+^ and related compounds.

In order to facilitate the evaluation of the results, most of the quantified compounds were grouped into five main groups: simple phenols and glycoside derivatives, secoiridoids and related compounds, flavonoids, lignans and triterpenic acids, as presented in Figure 1. Compounds not belonging to any of the mentioned chemical families are not included in the graphics but will be addressed individually in subsequent discussions. Figure 1 shows the distribution of the different groups of metabolites among the three analysed tissues for each cultivar. The sum concentration of all the compounds belonging to a given group is expressed as a percentage of the total amount found in the three matrices of each olive cultivar, which is normalized to 100. In this way, it is possible to depict several general tendencies, although, in some cases, great variability can be observed depending on the cultivar.

As far as simple phenols and glycoside derivatives were concerned, they were found in all three evaluated matrices. Roots were the matrix with the highest percentage of these types of compounds (40–70%), except for ‘Empeltre’, in which leaves were the richest plant organ with around 70% of the total. In the rest of the cultivars, leaves accounted for 20–40%, while the remaining 5–15% was found in stems. It is worth noticing that the distribution of individual metabolites belonging to this group was also diverse (Table 1); verbascoside and isoverbascoside were the only simple phenol glycosides found in roots; phenylethyl primeveroside was just detected in stems and hydroxytyrosol in leaves. Secoiridoids and related compounds were mostly found in leaves (45–60%), followed by stems (20–35%) and roots (10–30%). As previously mentioned, oleuropein was the secoiridoid found at the highest rates, ranging from 6736 mg kg^−1^ (in ‘Hojiblanca’ roots) to 74,453 mg kg^−1^ DW (in ‘Mastoidis’ leaves). Triterpenic acids was the other family of metabolites distributed throughout the three studied tissues. Between 65 and 75% of these compounds were found in leaves, followed by roots and stems with 5–20%, each. Oleanolic acid was the compound found at the highest concentration in leaves (10,152–15,670 mg kg^−1^ DW) and stems (1064–3775 mg kg^−1^ DW), while maslinic acid was the most concentrated in roots (2181–3897 mg kg^−1^ DW). No flavonoids were detected in roots, which agrees with previous works in which they were not found [27,41,53] or were reported at very low concentration levels [50,51]. Flavonoids were mostly found in leaves (80–90%), with minor amounts quantified in stems (510 to 1267 mg kg^−1^ DW). Moreover, as seen in Table 1, just the three isomers of luteolin *O*-glucoside and rutin were common to both matrices; quercetin *O*-glucoside, dihydroquercetin 3-*O*-glucoside, cyanidin *O*-glucoside, dihydrokaempferol and taxifolin were absent from leaves, and apigenin 7-*O*-glucoside, apigenin *O*-rutinoside, diosmin and gallocatechin were not found in stems. A comparable trend was observed for lignans. They were distributed homogeneously amongst roots (45–65%) and stems (35–55%) but were missing in leaves. Most of them were found in both matrices except for cycloolivil, which was only found in stems, and its glycosylated form, which appeared just in roots. Acetoxypinoresinol glucoside was the most abundant lignan in most cultivars, with contents ranging from 494 to 1588 mg kg^−1^ DW in stems, and 262 to 1001 mg kg^−1^ DW in roots. The absence of this family of compounds in olive leaves has been widely documented in other works dealing with the LC-MS phenolic profiling of leaves [41,54,55], while other authors have reported very low levels of lignans in this olive tissue [30,39,56]. 

Regarding the rest of the metabolites not belonging to any of the major groups, different behaviours can be pointed out. Quinic acid was quantified in the three matrices and presented the highest content in leaves (4439–7815 mg kg^−1^ DW), followed by stems (1469–2710 mg kg^−1^ DW) and roots (239–377 mg kg^−1^ DW). With respect to iridoids, they were not found in leaves and the highest content of these compounds was observed in roots, as previously described by Michel and collaborators for 7-deoxyloganic acid [41]. Finally, five unknown compounds were semi-quantified because of their high relative intensity in the profiles (the oleuropein calibration curve was used to quantify all of them). The highest contents were found for unknowns 2 and 5 in olive leaves. As already mentioned, no tentative identity was proposed for unknown 2, but unknown 5 could be an oleanolic acid derivative and its high relative abundance in leaves could support this hypothesis.

#### 3.2.2. Assessment of Differences in the Metabolic Profiles of the 10 Cultivars under Study

**Figure 2:**
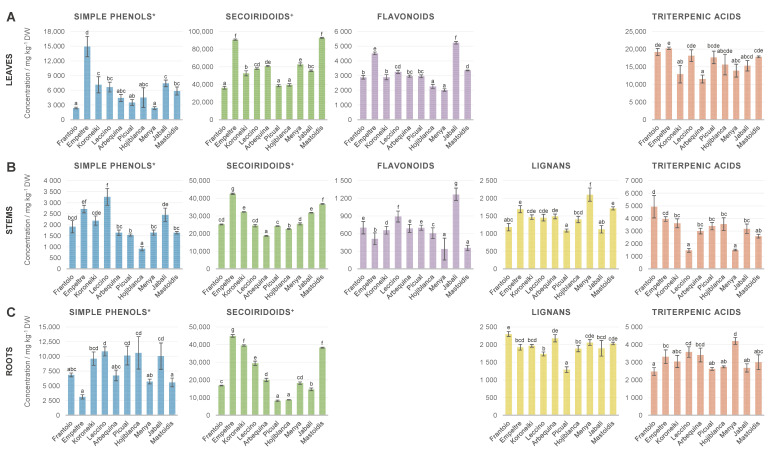
Sum concentration of the main metabolite classes in the three analysed tissues (leaves (**A**), stems (**B**) and roots (**C**)) of 10 different olive cultivars sorted by resistance to VWO: from HR (left) to ES (right)). Error bars show the standard deviation of three biological replicates (n = 3). Lower case letters indicate Tukey’s post hoc test differences (*p* < 0.05) among different cultivars. * and glycoside derivatives ^+^and related compounds.

It is widely recognized that the genetic origin is one of the main factors affecting the profile of secondary metabolites of olive-related matrices such as olive oil or olive leaves [28,39,48]. In this project, 10 different olive cultivars belonging to different resistance/susceptibility response categories to VWO, according to a previous evaluation of disease parameters, were studied [6,10]. Two cultivars belonging to each pre-existing category were chosen: ‘Frantoio’ and ‘Empeltre’ (HR); ‘Koroneiki and ‘Leccino’ (R); ‘Arbequina’ and ‘Picual’ (MS); ‘Hojiblanca’ and ‘Menya’ (S); ‘Jabali’ and ‘Mastoidis’ (ES). Thus, in a subsequent step, the generated quantitative data were re-evaluated with the final aim of finding possible links between the resistance to *V. dahliae* and the metabolic profiles of leaves, stems and roots of the cultivars under investigation. To facilitate the visualization of the results, Figure 2 presents the sum concentrations per compound class in the three olive tissues of each cultivar, following the same strategy of metabolite grouping as in Figure 1. It is important to note that the results shown are the average of three biological replicates (n = 3). Thus, the magnitude of the error bars illustrating the standard deviation makes complete sense if we have in mind the variability among different plant individuals. In general terms, the abundance of each group of metabolites in the different cultivars followed the same trend in the three plant tissues, i.e., the cultivar that exhibited the highest concentration of a specific group of compounds in one matrix also ranked among the richest ones in the other two matrices. This can be clearly observed for secoiridoids and related compounds, flavonoids, lignans and simple phenol and glycoside derivatives (except for roots). This indicates that the prevalence of secondary metabolites in the different tissues of the plant seems to be somehow proportional and is cultivar-dependent.

As discussed in the previous section, secoiridoids were the most abundant family of metabolites for all the evaluated cultivars. Indeed, they were one order of magnitude more abundant than the rest of groups of compounds, on average. Therefore, taking into account the contribution of all the quantified molecules, the general trend followed by secoiridoids is applicable to the total content of secondary metabolites. In this way, it is possible to deduce from Figure 2 that ‘Empeltre’ (HR) and ‘Mastoidis’ (ES) were the richest cultivars in terms of secoiridoids and, thus, in terms of secondary metabolites, while ‘Picual’ (MS), ‘Hojiblanca’ (S), ‘Arbequina’ (MS) and ‘Frantoio’ (HR) were among the cultivars with the lowest content of this fraction of compounds. These general observations suggest that the total amount of secondary metabolites does not correlate with the resistance/susceptibility of these olive cultivars to the fungus *V. dahliae*. This finding contrasts with the conclusions achieved by Gharbi and collaborators, that reported a higher total polyphenol content in roots and stems of ‘Sayali’ (resistant) compared to ‘Chemlali’ (extremely susceptible) olive trees [24]. However, it is important to note that a direct comparison of our results may not be entirely feasible. This discrepancy arises from the fact that these authors employed a colorimetric method to assess phenolic content in olive tissues and examined two olive cultivars that are not within the scope of our current study. Our working hypothesis suggests that the key factor contributing to a more effective protective response against the pathogen in resistant cultivars is likely linked to the compositional profile of plant organs rather than simply the total concentration of some families of metabolites.

In the case of secoiridoids, some cultivars stood out for presenting high amounts of specific metabolites, such as secologanoside in the three tissues of ‘Jabali’ (ES), or ligstroside in all ‘Leccino’ (R) samples (Table S2). Regarding simple phenols and glycoside derivatives, the highest contents were found in ‘Empeltre’ (HR) in leaves, and ‘Leccino’ and ‘Empeltre’ in stems (Figure 2). However, the prevalence of this family of metabolites in roots was completely different, and ‘Empeltre’ stood out for its low concentration. As far as flavonoids were concerned, ‘Jabali’ (ES) was the richest cultivar, followed by ‘Leccino’ in stems and ‘Empeltre’ in leaves, while ‘Menya’ (S) was among the cultivars with the lowest flavonoid content in both tissues. The compounds with the highest weight in the flavonoid profile were luteolin 7-*O*-glucoside in leaves, dihydrokaempferol in ‘Hojiblanca’ (S) stems and taxifolin in ‘Leccino’ (R) stems (Table S2). As seen in Figure 2, the contents of lignans were more homogeneous among the evaluated cultivars, although ‘Menya’ (S) could be pointed out as the richest variety in terms of this family of compounds in stems, while ‘Picual’ (MS) was the poorest in roots (statistical significance *p* < 0.05). Finally, triterpenic acids trends varied a lot from one kind of matrix to the others. For example, ‘Leccino’ and ‘Menya’ presented very low contents of these metabolites in stems, but they were among the richest cultivars in the other two matrices (Figure 2). ‘Arbequina’ (MS) stems presented a particular profile characterised by very similar amounts of maslinic and oleanolic acids, unlike the rest of the cultivars in which oleanolic acid was prevalent, as commented in Section 3.2.1.

Few prior studies have addressed the distribution of secondary metabolites across various olive plant tissues, and none of them have conducted such an investigation quantitatively. Consequently, there is no existing data to compare with our results.

### 3.3. Relationship between Cultivars Metabolic Profiles and Resistance/Susceptibility to the Soil Fungus Verticillium dahliae

To further investigate the possible relationship between tissues’ metabolic profiles and resistance to VWO, the quantitative results were evaluated by applying unsupervised and supervised multivariate analysis. In this way, 28 compounds in roots, 44 in stems and 34 in leaves were used as variables in the three data matrices (with 30 samples each) that were built for statistical analysis.

**Figure 3:**
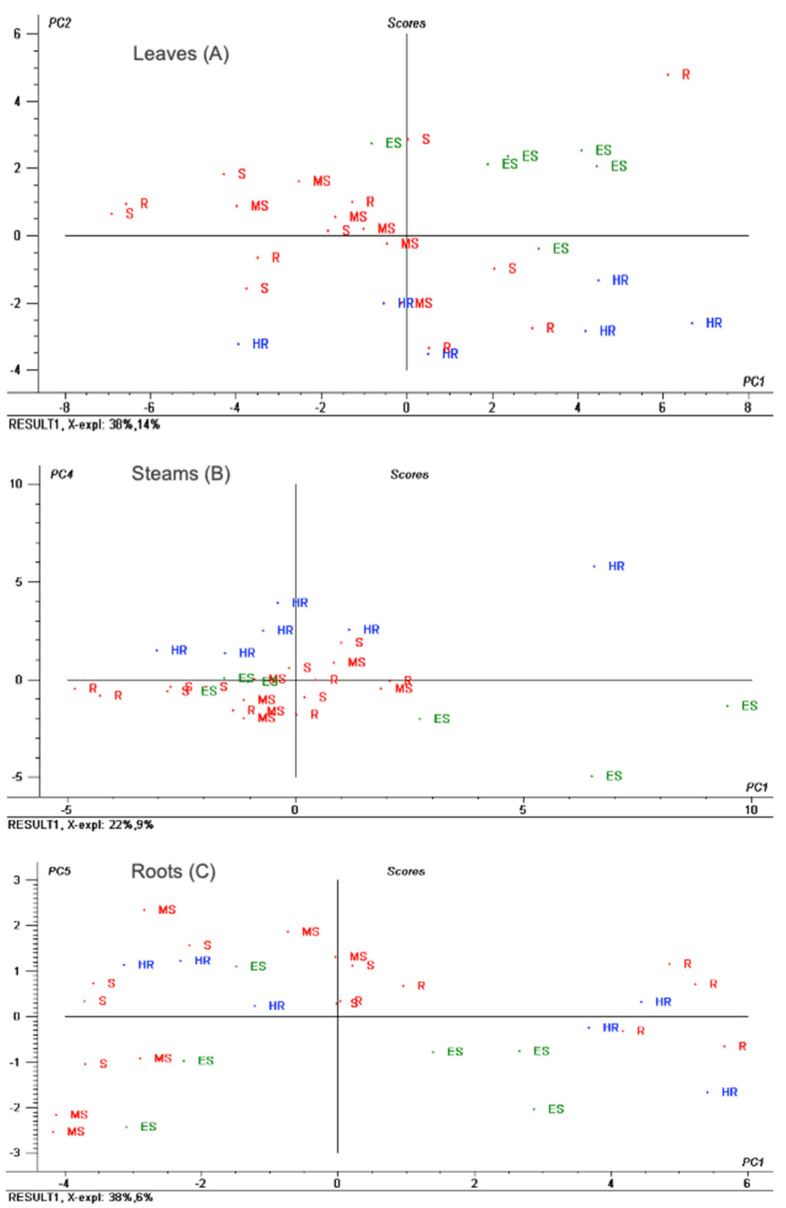
Principal component analysis (PCA) score plots representing the two principal components (PCs) showing best samples’ grouping for each olive tissue: (**A**) leaves (PC1 vs. PC2), (**B**) stems (PC1 vs. PC4) and (**C**) roots (PC1 vs. PC5).

Initially, principal component analysis (PCA) was performed to check the natural clustering of samples from each tissue type. Figure 3 shows the obtained PCA score plots for the two principal components (PCs) showing the best grouping of the samples for each olive tissue. In the leaf samples, the first two PCs covered 52% of the variance, and discriminated between HR (blue colour) and ES (green colour) cultivars along the PC2. The rest of the categories (MS, S and R), coloured in red, were mixed in the central cluster (Figure 3A). Score values for PC2 were high for the ES category, which means that compounds with positive loadings (Figure S1A) such as elenolic acid glucoside (isomers 1 and 2), unknown 1, hydroxytyrosol glucoside and neonuzhenide/oleuropein glucoside (isomer 3) were positively related to ES cultivars. The negative loadings (Figure S1A) corresponded to unknown 5, betulinic acid, oleuropein aglycone (isomer 2), oleanolic acid, neonuzhenide/oleuropein glucoside (isomer 4), maslinic acid and quinic acid, among others. According to scores for PC2, these metabolites were positively related to the HR category. The loading plot also revealed the importance of unknown 2 for the separation of some leaf samples with intermediate susceptibility (Figure S1A). In stem samples, the first two PCs explained 40% of the variance. However, PC4, explaining only 9% of the variance, was responsible for a moderately good separation among HR, intermediate susceptibility/resistance (MS, S and R) and ES categories (Figure 3B). In this case, the loading plot (Figure S1B) indicated that the main compounds affecting the separation were oleanolic acid, quinic acid, acetoxypinoresinol, unknown 5 and oleuropein aglycone (isomer 1) (positive loadings showing a positive relation with the HR category), and neonuzhenide/oleuropein glucoside (isomer 2), unknown 2, elenolic acid glucoside (isomer 2), hydroxytyrosol glucoside, cyanidin *O*-glucoside, quercetin *O*-glucoside (isomers 1 and 3), and the aldehydic form of decarboxymethyl elenolic acid glucoside (negative loadings showing a positive relation with ES cultivars). Lastly, in root samples, the first two PCs covered 53% of the variance, but good separation among cultivars from different resistance categories could barely be found (data not shown). PC5, explaining just 6% of the variance, provided a slightly better separation between the HR and ES categories (Figure 3C). The loading plot (Figure S1C) showed that compounds such as acetoxypinoresinol, acetoxypinoresinol glucoside, oleanolic acid, betulinic acid, hydroxypinoresinol glucoside and maslinic acid presented a positive relation with HR cultivars and some samples from the intermediate susceptibility categories (which were quite mixed in this case). On the contrary, negative loadings showed as main compounds: isoverbascoside, hydroxyoleuropein, lucidumoside C and unknown 3, with a positive relation to ES cultivars and a negative one to the HR category. Overall, none of the quantitative data from the three studied olive tissues pointed out any clear potential marker for cultivars with intermediate resistance.

In a later stage, supervised partial least squares discriminant analysis (PLS-DA) was performed to discriminate samples belonging to olive cultivars showing different resistance/susceptibility to VWO. This time, cultivars were grouped in the three classes inferred from the previous unsupervised analysis: HR (class 1), MS + S + R (class 2) and ES (class 3). The full cross-validation parameters of the models built for each olive tissue type are displayed in Table 2. Confusion matrices showing correctly and wrongly classified samples from the cross-validation subset can be also found in Table 2. As already seen in the PCA plots (Figure 3), the metabolites from the matrix showing less capability to discriminate samples among resistance categories were those from the roots. The root PLS-DA model presented the lowest accuracy and the highest error rate. In fact, it showed the worst classification capacity, with 10 samples assigned to a wrong class (Table 2). On the other hand, both leaf and stem models presented very satisfactory correct classification rates, showing accuracy values of 90 and 93% for cross-validation, and only 3 and 2 wrongly classified samples, respectively. Interestingly, in both tissues, HR and ES categories were well-classified and those samples wrongly classified corresponded to cultivars with intermediate susceptibility (class 2). This might be due to the fact that the latter was the broader class, which, as mentioned before, gathered together three intermediate resistance categories (MS, S and R), presenting very diverse metabolic profiles.

The variables’ influence in each class of the three PLS-DA models is depicted in Figure S2, which shows the joint representation of the regression coefficient of each metabolite and its variable importance on the projection (VIP) value. Compounds with VIP values higher than 1 could be pointed out as potential markers for each class (in this case a VIP value > 1.2–1.5 was considered). It is possible to see that they correspond to minimum and maximum regression coefficients, which give an idea of the importance of each variable in the prediction. Thus, the information retrieved from these graphs can be used to describe typical compositional patterns of cultivars belonging to distinct categories of resistance/susceptibility for each studied olive tissue (Table 3). As expected, some of these potential markers were also the variables influencing the most sample clustering in the PCA models (Figure 3 and Figure S1), such as maslinic acid, oleuropein aglycone (isomer 2), hydroxytyrosol glucoside and elenolic acid glucoside (isomers 1 and 2) in leaves; quinic acid, acetoxypinoresinol, unknown 5 and neonuzhenide/oleuropein glucoside (isomer 2) in stems; and betulinic acid in roots. It is also worth noting that some metabolites appeared as markers of the same category in different olive tissues, which reinforcers their discriminating role in the metabolic profiles. For example, low levels of elenolic acid glucoside (isomer 2) were characteristic features of HR cultivars both in leaves and stems, showing high VIP values and negative regression coefficients. A high content of 11-hydroxyiridodial glucoside pentaacetate was characteristic of stems and roots of cultivars with medium susceptibility to VWO, while the opposite behaviour was typical from the same tissues from ES cultivars. In the same way, high levels of maslinic acid were found in leaves and stems of ES cultivars, while cultivars with medium susceptibility were characterized by low levels of this triterpenic acid.

When just one tissue is considered, contrasting trends of some metabolites in different categories can be observed. This means that it is the synergistic effect of several metabolites (described as compositional pattern) which could be linked to the level of resistance to VWO. For example, low levels of maslinic acid were found in leaves of cultivars belonging to the medium susceptibility class, while high levels were typical of HR cultivars when accompanied by a high concentration of oleuropein aglycone (isomer 2), and low contents of lucidumoside C, oleuroside, neonuzhenide/oleuropein glucoside (isomer 3), hydroxytyrosol glucoside and elenolic acid glucoside (isomers 1 and 2). On the contrary, high leaf levels of maslinic acid and the aforementioned metabolites (lucidumoside C, oleuroside, neonuzhenide/oleuropein glucoside (isomer 3), hydroxytyrosol glucoside and elenolic acid glucoside (isomers 1 and 2)), together with low levels of oleuropein aglycone (isomer 2) were typical from ES cultivars. In stems, neonuzhenide/oleuropein glucoside (isomer 2) could be pointed out as a specific marker with a contrary trend in the extreme categories: low content in HR cultivars (negative regression coefficient) and high concentration in ES samples (positive regression coefficient). Moreover, stems from ES cultivars presented low concentrations of 11-hydroxyiridodial glucoside pentaacetate and high levels of oleuropein, oleuroside, metoxypinoresinol glucoside and two triterpenic acids (betulinic and maslinic), exactly the opposite pattern shown by samples from the medium susceptibility categories. Finally, in roots, high concentrations of cycloolivil glucoside (isomer 2) and betulinic acid were characteristic of HR cultivars, while a high content of betulinic acid and low cycloolivil glucoside (isomer 2) concentration were typical from medium susceptibility cultivars, and low levels of both metabolites were representative of the ES category. 11-hydroxyiridodial glucoside pentaacetate and hydroxypinoresinol glucoside were also useful to discriminate cultivars from the medium susceptibility class, where they were found at high concentrations (positive correlation coefficients), and ES cultivars, which presented low levels of these two compounds (negative correlation coefficients).

The description of these compositional patterns in cultivars displaying varying levels of resistance or susceptibility to VWO stands out as a major accomplishment of this research. This information holds the potential for categorising olive cultivars in the future based on the metabolic profiles of their leaves, stems or roots. Furthermore, it is noteworthy to highlight the impressive results generated through the statistical analysis, particularly given the significant diversity among the samples under study, which encompassed 10 different olive cultivars classified into 5 resistance/susceptibility categories. Extending this study with additional cultivars and replicates in future projects presents a promising prospect for future research. 

## 4. Conclusions

In this work, the metabolic profiles of leaves, stems and roots of 10 different olive cultivars with different degrees of resistance/susceptibility to VWO were studied by applying a multiclass LC-MS method (using both high- and low-resolution analysers with qualitative and quantitative purposes, respectively). A total of 56 compounds belonging to several chemical classes (organic acids, simple phenols, secoiridoids, flavonoids, lignans, triterpenic acids, etc.) were identified in the profiles. From them, 28 were quantified in roots, 44 in stems and 34 in leaves, and their distribution among the three tissues was established. In general, although no flavonoids were found in roots and no lignans were detected in leaves, the prevalence of the chemical families found commonly throughout the different plant organs seemed to be consistent and cultivar-dependent. PCA and PLS-DA were also performed on the quantitative data matrices of the evaluated olive tissues to investigate the possible relationship between the metabolite content and the cultivar’s susceptibility level, trying to gain a deeper understanding of the metabolic processes underlying olive resistance to VWO. The models for both leaves and stems exhibited highly commendable correct classification rates, achieving accuracy values of 90% and 93% for cross-validation, respectively. Our findings revealed that cultivars showing similar susceptibility levels shared common compositional patterns. This discovery holds the potential to facilitate the identification of optimal genitor candidates in future breeding programs, aiming to develop cultivars with heightened resistance to VWO while maintaining favourable agronomic characteristics. Furthermore, the models, constructed using the information from various olive tissues, consistently underscored certain compounds as potential markers of resistance and susceptibility, suggesting their possible involvement in the plant’s defence mechanisms against *V. dahliae*. For instance, the levels of elenolic acid glucoside (isomer 2) and the aldehydic form of decarboxymethyl elenolic acid glucoside in leaves and stems exhibited an inverse correlation with VWO resistance. A similar negative correlation was established for VWO susceptibility and the contents of 11-hydroxyiridodial glucoside pentaacetate in stems and roots. In addition, high concentrations of maslinic acid in leaves and stems were linked to higher susceptibility to VWO. In this way, a targeted quantification of such specific metabolites could serve as a valuable tool for predicting resistance/susceptibility of new genotypes from crossbreeding. 

## Figures and Tables

**Table 2:** Cross-validation confusion matrices and validation parameters for the PLS-DA classification models. Class 1: highly resistant cultivars; class 2: resistant, moderately susceptible and susceptible cultivars; class 3: extremely susceptible cultivars.

	Leaves			Stems			Roots		
Real/Predicted	Class 1	Class 2	Class 3	Class 1	Class 2	Class 3	Class 1	Class 2	Class 3
Class 1	6	0	0	6	0	0	5	0	1
Class 2	2	15	1	2	16	0	5	9	4
Class 3	0	0	6	0	0	6	0	0	6
Components		3			4			3	
Error rate		0.06			0.04			0.28	
Accuracy		0.90			0.93			0.70	

**Table 3:** Compositional patterns of cultivars belonging to different resistance/susceptibility categories as pointed out by the PLS-DA models built for each olive tissue type.

		Highly Resistant Cultivars			Medium Susceptibility Cultivars *			Extremely Susceptible Cultivars		
		Metabolite	Regression Coefficient	VIP Value	Metabolite	Regression Coefficient	VIP Value	Metabolite	Regression Coefficient	VIP Value
Leaves	**↑**	Maslinic acid	0.057	1.60	Gallocatechin	0.121	3.78	Lucidumoside C	0.064	1.43
		Oleuropein aglycone (is 2)	0.055	1.92				Oleuroside	0.060	1.44
								Neonuzhenide/oleuropein glucoside (is 3)	0.060	2.05
								Hydroxytyrosol glucoside	0.057	1.87
								Elenolic acid glucoside (is 2)	0.038	1.71
								Maslinic acid ^+^	0.035	1.82
								Elenolic acid glucoside (is 1)	0.019	1.11
	**↓**	Lucidumoside C	−0.002	1.35	Luteolin 7-*O*-glucoside (is 1)	−0.066	1.65	Oleuropein aglycone (is 2)	−0.001	1.49
		Oleuroside	−0.007	1.61	Chrysoeriol *O*-glucoside	−0.070	2.03	Aldehydic form of DEA glucoside	−0.004	1.37
		Neonuzhenide/oleuropein glucoside (is 3)	−0.030	2.83	Oleuropein aglycone (is 1)	−0.077	2.05			
		Hydroxytyrosol glucoside	−0.032	2.62	Demethyl oleuropein	−0.084	2.19			
		Elenolic acid glucoside (is1)	−0.040	1.53	Maslinic acid ^+^	−0.092	2.96			
		Elenolic acid glucoside (is 2) ^+^	−0.045	2.54						
		Aldehydic form of DEA glucoside ^+^	−0.053	1.49						
Stems	**↑**	Unknown 4	0.058	3.39	11-Hydroxyiridodial glucoside pentaacetate ^+^	0.096	2.12	Oleuroside	0.094	4.04
		Quinic acid	0.051	2.49				Metoxypinoresinol glucoside	0.067	2.14
		Demethyl oleuropein	0.042	1.94				Oleuropein	0.060	3.34
		Oleanolic acid	0.036	2.70				Neonuzhenide/oleuropein glucoside (is 2)	0.057	1.65
		Acetoxypinoresinol	0.029	1.64				Betulinic acid	0.045	1.68
		Unknown 5	0.022	2.06				Maslinic acid ^+^	0.023	1.63
	**↓**	Neonuzhenide/oleuropein glucoside (is 2)	−0.029	2.55	Betulinic acid	−0.059	2.21	11-Hydroxyiridodial glucoside pentaacetate ^+^	−0.085	1.90
		Aldehydic form of DEA glucoside ^+^	−0.038	1.54	Maslinic acid ^+^	−0.063	1.92			
		Elenolic acid glucoside (is 2) ^+^	−0.042	2.40	Metoxypinoresinol glucoside	−0.086	2.71			
		Unknown 2	−0.045	2.96	Oleuropein	−0.112	4.08			
					Oleuroside	−0.130	5.18			
Roots	**↑**	Cycloolivil glucoside (is 2)	0.082	5.69	11-Hydroxyiridodial glucoside pentaacetate ^+^	0.110	2.56	Acetoxypinoresinol glucoside	0.109	3.04
		Betulinic acid	0.047	1.94	Hydroxypinoresinol glucoside	0.092	1.92	Unknown 1	0.083	1.74
		Elenolic acid glucoside (is 1)	0.034	1.42	Betulinic acid	0.052	1.70			
	**↓**	Verbascoside	−0.047	1.75	Acetoxypinoresinol glucoside	−0.110	2.20	Cycloolivil glucoside (is 2)	−0.015	1.67
					Cycloolivil glucoside (is 2)	−0.067	2.56	Oleanolic acid	−0.076	2.17
								Hydroxypinoresinol glucoside	−0.088	2.78
								Betulinic acid	−0.099	3.06
								11-Hydroxyiridodial glucoside pentaacetate ^+^	−0.113	3.85

* including resistant, moderately susceptible and susceptible cultivars; ^+^ markers of a given category matching in several olive tissues. Abbreviations: ↑, high content; ↓, low content; is, isomer; DEA, decarboxymethyl elenolic acid.

## Data Availability

The data presented in this study are available in Table S2.

## Supplementary Materials

The following supporting information can be downloaded at: https://www.mdpi.com/article/10.3390/antiox12122120/s1, Table S1: Analytical parameters of the LC-IT MS method; Table S2: Quantitative results obtained by LC-IT MS. Results expressed as mg kg^−1^ of olive tissue DW ± standard deviation (leaves, a; stems, b; roots, c); Figure S1: Principal component analysis (PCA) loading plots representing the two principal components (PCs) showing the best samples’ grouping for each olive tissue: leaves (A), stems (B) and roots (C); Figure S2: Representation of the regression coefficients and variable importance on the projection (VIP) values of each metabolite quantified in the three olive tissues under study: leaves (A), stems (B) and roots (C).
