# Supplementary material for: Characterization of the Metabolic Profile of Olive Tissues (Roots, Stems and Leaves): Relationship with Cultivars’ Resistance/Susceptibility to the Soil Fungus Verticillium dahliae
Source: Antioxidants (Basel). 2023 Dec 15;12(12):2120. doi: 10.3390/antiox12122120 (PMC10741231; doi:10.3390/antiox12122120)
Supplement: Supplementary file 1 [file antioxidants-12-02120-s001.zip › antioxidants-2751761-supplementary.pdf]

Supplementary Material

Characterization of the Metabolic Profile of Olive Tissues (Roots, Stems and Leaves): Relationship with Cultivars' Resistance/Susceptibility to the Soil Fungus *Verticillium dahliae*

Irene Serrano-García[1], Lucía Olmo-García[1], Olga Monago-Maraña[2], Iván Muñoz Cabello de Alba[1], Lorenzo León[3], Raúl de la Rosa[3], Alicia Serrano[4], Ana María Gómez-Caravaca[1], Alegría Carrasco-Pancorbo[1]

[1]Department of Analytical Chemistry, Faculty of Sciences, University of Granada. Ave. Fuentenueva s/n, E-18071 Granada, Spain.
[2]Department of Analytical Sciences, Faculty of Sciences, Universidad Nacional de Educación a Distancia (UNED), Avda. Esparta s/n, Crta. de Las Rozas-Madrid, E-28232 Madrid, Spain.
[3]Instituto de Investigación y Formación Agraria y Pesquera (IFAPA), Centro Alameda del Obispo, Ave. Menéndez Pidal s/n, E-14004, Córdoba, Spain.
[4]Department of Experimental Biology. The University Institute of Research on Olive and Olive Oils (INUO). University of Jaén. Campus Las Lagunillas s/n, 23071 Jaén, Spain.

Index:

Table S1. Analytical parameters of the LC-IT MS method. *(pag 2)*

Table S2. Quantitative results obtained by LC-IT MS. Data expressed as mg kg^{-1} of olive tissue (DW) ± standard deviation (leaves, a; stems, b; roots, c). *(pag 3)*

Figure S1. Principal component analysis (PCA) loadings plots representing the two principal components (PCs) showing the best samples' grouping for each olive tissue: leaves (A), stems (B) and roots (C). *(pag 6)*

Figure S2. Representation of the regression coefficients and variable importance on the projection (VIP) values of each metabolite quantified in the three olive tissues under study: leaves (A), stems (B) and roots (C). *(pag 7)*

Compound numbers in Figures S1 and S2. *(pag 8)*

Table S1. Analytical parameters of the LC-IT MS method.

Compound	Calibration curve	R^2	Dynamic linear range / mg l^{-1}	LOD[a] / µg l^{-1}	LOQ[a] / µg l^{-1}	*Intra*-day repeatability[b] (area RSD / %)	*Inter*-day repeatability[c] (area RSD / %)
Quinic acid	y = 6212.5 + 36428.1x<br>y = 512246.7 + 17005.7x	0.9983<br>0.9948	LOQ – 18.7<br>18.7 – 150.0	24.4	81.3	0.33	0.49
Hydroxytyrosol	y = 8449.0 + 31061.1x	0.9976	LOQ – 52.0	47.3	157.6	7.08	10.62
Tyrosol	y = -583.7 + 10334.9x<br>y = 107822.0 + 6833.5x	0.9997<br>0.9898	LOQ – 25.0<br>25.0 – 100.0	171.3	571.0	5.70	8.56
Rutin	y = -36097.3 + 169422.6x<br>y = 1067657.8 + 107842.2x	0.9992<br>0.9911	LOQ – 12.0<br>12.0 – 96.0	28.4	94.6	5.23	7.85
Luteolin-7-*O*-glucoside	y = 56511.5 + 411020.5x<br>y = 2348016.3 + 193405.8x	0.9966<br>0.9924	LOQ – 8.0<br>8.0 – 64.0	18.7	62.3	1.66	2.49
Verbascoside	y = 53328.5 + 111178.1x	0.9955	LOQ – 52	42.1	140.2	3.63	5.44
Oleuropein	y = 514056.1 + 183004.9x<br>y = 10361638.0 + 48313.3x	0.9942<br>0.9954	LOQ – 62.5<br>62.5 – 500.0	25.4	84.6	2.25	3.37
Maslinic acid	y = 166450.8 + 197117.1x<br>y = 2244039.3 + 54184.3x	0.9899<br>0.9933	LOQ – 12.5<br>12.5 – 100.0	14.9	49.5	3.59	5.38
Betulinic acid	y = 70030.7 + 930415.0x<br>y = 1436593.5 + 396367.1x	0.9916<br>0.9902	LOQ – 2.5<br>2.5 – 10.0	2.0	6.7	1.36	2.05
Oleanolic acid	y = 251665.6 + 430573.6x<br>y = 2409833.3 + 151759.4x	0.9913<br>0.9949	LOQ – 9.0<br>9.0 – 72.0	5.8	19.2	1.96	2.94

[a] Calculated as the concentration that generates a signal to noise ratio equal to 3 (LOD) and 10 (LOQ).
[b] RSD (relative standard deviation) of peak area for 6 injections of the quality control sample carried out within the same sequence.
[c] RSD of peak area for 20 injections of the quality control sample from different sequences carried out over 3 days.
Abbreviations: LOD, Limit of detection; LOQ, Limit of quantification.

Table S2. Quantitative results obtained by LC-IT MS. Data expressed as mg kg^{-1} of olive tissue (DW) ± standard deviation.

a) Leaves	*Arbequina*	*Empeltre*	*Frantoio*	*Hojiblanca*	*Jabali*	*Koroneiki*	*Leccino*	*Mastoidis*	*Menya*	*Picual*
Aldehydic form of decarboxymethyl elenolic acid glucoside	771±156^{ab}	741±132^{ab}	240±77^{a}	1095±670^{abc}	1891±374^{c}	957±558^{abc}	648±133^{ab}	438±35^{ab}	1274±411^{bc}	1239±287^{abc}
Apigenin-7-O-glucoside	138±21^{a}	193±34^{a}	234±80^{a}	250±198^{a}	422±82^{a}	80±43^{a}	1025±364^{b}	236±23^{a}	353±109^{a}	246±81^{a}
Apigenin-O-rutinoside	436±61^{ab}	404±50^{ab}	270±76^{ab}	411±196^{ab}	564±165^{bc}	142±51^{a}	854±251^{c}	288±39^{ab}	445±113^{ab}	328±58^{ab}
Betulinic acid	113±33^{ab}	234±19^{b}	165±25^{ab}	151±77^{ab}	149±57^{ab}	118±57^{ab}	181±49^{ab}	197±20^{ab}	96±31^{a}	137±56^{ab}
Chrysoeriol-O-glucoside	82±20^{a}	209±54^{a}	205±85^{a}	81±59^{a}	173±30^{a}	129±85^{a}	117±51^{a}	167±17^{a}	65±9^{a}	103±35^{a}
Demethyl oleuropein	510±182^{a}	1053±69^{ab}	826±329^{ab}	445±270^{a}	2021±1240^{b}	591±217^{a}	316±127^{a}	598±101^{a}	329±201^{a}	587±472^{a}
Diosmin	222±84^{a}	682±195^{b}	114±34^{a}	127±127^{a}	308±105^{a}	231±123^{a}	147±83^{a}	259±79^{a}	57±21^{a}	133±50^{a}
Elenolic acid glucoside (isomer 1)	506±67 ^{a}	358±30^{a}	334±127^{a}	297±112^{a}	510±120^{a}	464±306^{a}	398±157^{a}	680±111^{a}	596±147^{a}	314±48^{a}
Elenolic acid glucoside (isomer 2)	227±9^{a}	303±71^{a}	158±54^{a}	338±191^{a}	609±49^{abc}	904±478^{bc}	281±68^{a}	1035±182^{c}	381±103^{ab}	485±176^{ab}
Gallocatechin	46±13^{a}	48±6^{ab}	30±9^{a}	59±19^{ab}	29±7^{a}	40±16^{a}	37±5^{a}	51±6^{ab}	59±9^{ab}	80±14^{b}
Hydroxytyrosol	577±107^{ab}	725±38^{ab}	726±243^{ab}	354±123^{a}	714±61^{ab}	585±304^{ab}	786±134^{ab}	880±54^{b}	479±132^{ab}	492±156^{ab}
Hydroxytyrosol glucoside	1241±141^{ab}	1347±254^{ab}	450±102^{a}	649±509^{ab}	3678±1037^{c}	2872±2071^{bc}	1760±563^{abc}	2845±114^{bc}	1209±434^{ab}	811±187^{ab}
Ligstroside	3991±728^{ab}	8608±1434^{c}	2085±808^{a}	2136±368^{a}	3320±920^{ab}	3393±2351^{ab}	3732±1531^{ab}	6372±1012^{bc}	3507±1139^{ab}	2292±959^{a}
Lucidumoside C	89±16^{a}	100±14^{a}	177±57^{abc}	88±37^{a}	212±92^{abc}	116±54^{ab}	110±38^{a}	264±62^{c}	253±14^{bc}	107±41^{a}
Luteolin-7-O-glucoside (isomer 1)	1263±353^{ab}	1726±427^{ab}	1430±677^{ab}	720±660^{a}	2674±432^{b}	1175±899^{ab}	768±414^{a}	1347±128^{ab}	586±464^{a}	1265±498^{ab}
Luteolin-O-glucoside (isomer 2)	403±145^{a}	530±175^{a}	375±181^{a}	411±356^{a}	508±71^{a}	454±343^{a}	129±61^{a}	447±34^{a}	183±35^{a}	450±197^{a}
Luteolin-O-glucoside (isomer 3)	86±17^{ab}	144±45^{ab}	59±18^{a}	62±45^{a}	94±26^{ab}	145±87^{ab}	39±18^{a}	189±23^{b}	83±23^{ab}	111±38^{ab}
Maslinic acid	1274±254^{a}	4768±186^{c}	3368±288^{bc}	2039±984^{ab}	3292±925^{bc}	2047±892^{ab}	2342±784^{ab}	3430±26^{bc}	1580±539^{ab}	2559±908^{ab}
Neonuzhenide/oleuropein glucoside (isomer 1)	48±19^{a}	58±5^{a}	29±10^{a}	72±51^{a}	174±83^{a}	177±148^{a}	180±112^{a}	62±11^{a}	615±405^{a}	57±19^{a}
Neonuzhenide/oleuropein glucoside (isomer 3)	416±56^{ab}	501±29^{ab}	228±84^{a}	272±178^{a}	686±122^{bc}	438±240^{ab}	489±159^{ab}	899±88^{c}	407±152^{ab}	393±114^{ab}
Neonuzhenide/oleuropein glucoside (isomer 4)	163±44^{a}	157±15^{a}	520±204^{b}	191±83^{a}	106±21^{a}	75±35^{a}	257±100^{ab}	242±26^{a}	214±120^{a}	245±113^{a}
Oleanolic acid	10152±2241^{a}	15255±632^{a}	15670±1969^{a}	13431±5751^{a}	11844±2796^{a}	10749±4897^{a}	15636±3204^{a}	14196±504^{a}	12239±3635^{a}	14957±3449^{a}
Oleoside/secologanoside (isomer 1)	626±95^{abc}	1095±135^{d}	382±108^{a}	566±151^{abc}	682±55^{abc}	625±179^{abc}	673±55^{abc}	893±52^{cd}	818±198^{bcd}	523±138^{ab}
Oleoside/secologanoside (isomer 2)	1666±402^{ab}	3515±316^{c}	1005±360^{a}	1465±781^{ab}	2347±313^{abc}	1502±702^{ab}	1990±229^{ab}	1551±28^{ab}	2580±942^{bc}	1541±101^{ab}
Oleuropein	49364±4286^{abc}	70692±5619^{bc}	28663±12486^{a}	31231±10647^{a}	40919±6365^{ab}	42112±21892^{ab}	46581±7692^{abc}	74453±5306^{c}	50140±13178^{abc}	29239±9434^{a}
Oleuropein aglycone (isomer 1)	203±83^{a}	640±160^{b}	263±41^{a}	124±47^{a}	124±71^{a}	101±61^{a}	190±86^{a}	572±205^{b}	74±12^{a}	164±86^{a}
Oleuropein aglycone (isomer 2)	95±12^{ab}	391±59^{d}	325±50^{cd}	130±62^{abc}	78±32^{a}	99±59^{ab}	280±132^{bcd}	317±123^{cd}	71±20^{a}	104±9^{ab}
Oleuroside	2147±277^{bc}	2567±106^{c}	999±477^{a}	1102±426^{ab}	1928±214^{abc}	1093±561^{ab}	1666±476^{abc}	4349±403^{d}	1469±458^{abc}	1309±327^{ab}
Quinic acid	5802±590^{abc}	7815±328^{c}	6357±1190^{abc}	4992±1146^{ab}	5916±463^{abc}	5826±1589^{abc}	7229±1019^{bc}	5549±350^{abc}	4769±251^{ab}	4439±574^{a}
Rutin	287±132^{a}	578±159^{a}	166±25^{a}	145±130^{a}	461±144^{a}	493±368^{a}	142±52^{a}	368±60^{a}	188±70^{a}	240±162^{a}
Unknown 1	120±60^{a}	42±14^{a}	29±7^{a}	56±10^{a}	107±46^{a}	260±242^{a}	81±58^{a}	265±43^{a}	113±51^{a}	24±15^{a}
Unknown 2	423±39^{ab}	258±47^{a}	440±59^{ab}	488±127^{b}	402±64^{ab}	409±99^{ab}	351±70^{ab}	461±80^{ab}	545±66^{b}	458±80^{ab}
Unknown 5	763±155^{ab}	958±39^{b}	813±218^{ab}	588±248^{ab}	386±97^{a}	546±200^{ab}	935±279^{b}	823±46^{ab}	585±170^{ab}	569±190^{ab}
Verbascoside	2638±1403^{a}	12863±4150^{b}	1204±221^{a}	3506±3379^{a}	3025±853^{a}	3676±2519^{a}	4118±1928^{a}	2137±1623^{a}	706±316^{a}	2216±1177^{a}

Lower case letters indicate Tukey's post hoc test differences ($p<0.05$) among different cultivars. Means with a common letter are not significantly different ($p>0.05$)

Table S2. Quantitative results obtained by LC-IT MS. Data expressed as mg kg^{-1} of olive tissue (DW) ± standard deviation *(cont.)*.

b) Stems	*Arbequina*	*Empeltre*	*Frantoio*	*Hojiblanca*	*Jabali*	*Koroneiki*	*Leccino*	*Mastoidis*	*Menya*	*Picual*
11-Hydroxyiridodial glucoside pentaacetate	15±3^{a}	81±23^{bcd}	36±4^{ab}	27±4^{ab}	18±5^{a}	94±20^{cd}	105±47^{d}	7±1^{a}	51±17^{abc}	22±8^{a}
Acetoxipinoresinol	112±26^{bcd}	143±57^{d}	70±27^{abc}	96±17^{abcd}	44±8^{ab}	50±8^{abc}	57±4^{abc}	69±14^{abc}	116±25^{cd}	31±6^{a}
Acetoxypinoresinol glucoside	1052±133^{bc}	1172±189^{cd}	613±118^{ab}	996±179^{abc}	779±207^{abc}	1102±131^{bcd}	637±24^{ab}	1268±85^{cd}	1588±365^{d}	494±80^{a}
Aldehydic form of decarboxymethyl elenolic acid glucoside	286±9^{a}	397±11^{ab}	220±54^{a}	678±66^{c}	1114±236^{d}	747±11^{c}	168±30^{a}	245±18^{a}	294±36^{a}	640±126^{bc}
Betulinic acid	76±18^{ab}	128±15^{abc}	176±52^{cd}	133±28^{bc}	211±29^{d}	222±19^{d}	58±16^{a}	184±10^{cd}	118±23^{abc}	81±15^{ab}
Cyanidin-O-glucoside	18±3^{a}	23±6^{a}	16±3^{a}	101±15^{b}	88±33^{b}	13±1^{a}	92±38^{b}	16±1^{a}	6±2^{a}	76±6^{b}
Cycloolivil	80±7^{ab}	137±21^{ab}	273±171^{ab}	74±16^{a}	101±4^{ab}	108±9^{ab}	296±148^{b}	81±19^{ab}	173±61^{ab}	87±13^{ab}
Demethyl oleuropein	42±10^{abc}	128±38^{d}	76±23^{abcd}	32±9^{ab}	96±17^{cd}	70±15^{abcd}	93±40^{bcd}	37±5^{abc}	87±13^{abcd}	28±4^{a}
Dihydrokaempferol	163±10^{bc}	116±21^{b}	60±3^{a}	278±27^{e}	200±23^{cd}	59±7^{a}	188±31^{cd}	45±7^{a}	31±5^{a}	229±12^{de}
Dihydroquercetin 3-O-glucoside	62±12^{ab}	43±4^{ab}	84±32^{abc}	20±4^{a}	142±43^{cd}	103±3^{bcd}	177±56^{d}	76±4^{abc}	51±7^{ab}	76±21^{abc}
Elenolic acid glucoside (isomer 1)	308±22^{a}	555±74^{b}	328±55^{a}	383±20^{ab}	558±15^{b}	419±42^{ab}	427±143^{ab}	397±36^{ab}	481±93^{ab}	345±30^{a}
Elenolic acid glucoside (isomer 2)	81±11^{a}	182±48^{abc}	71±26^{a}	92±21^{ab}	278±31^{cde}	237±36^{bcd}	402±125^{e}	352±53^{de}	315±53^{cde}	196±33^{abc}
Hydroxy oleuropein	126±42^{ab}	146±33^{ab}	94±42^{a}	158±31^{ab}	148±28^{ab}	133±67^{ab}	80±30^{a}	174±40^{ab}	221±62^{b}	119±18^{ab}
Hydroxypinoresinol glucoside	105±11^{a}	125±34^{a}	90±18^{a}	146±11^{a}	66±10^{a}	77±4^{a}	356±110^{b}	148±18^{a}	106±27^{a}	368±28^{b}
Hydroxytyrosol glucoside	1273±222^{bc}	1472±278^{c}	767±32^{ab}	373±126^{a}	1545±428^{c}	1610±379^{c}	1301±162^{bc}	1350±94^{bc}	954±138^{abc}	1010±47^{abc}
Ligstroside	957±169^{a}	2885±388^{bc}	1345±151^{abc}	1402±32^{abc}	1623±509^{abc}	1311±343^{ab}	2916±1403^{c}	1905±431^{abc}	1599±127^{abc}	2145±464^{abc}
Lucidumoside C	242±117^{abc}	200±33^{ab}	108±54^{a}	247±30^{abc}	216±74^{ab}	246±143^{abc}	103±33^{a}	357±105^{bc}	485±123^{c}	147±37^{ab}
Luteolin-7-O-glucoside (isomer 1)	36±16^{a}	12±1^{a}	51±36^{ab}	21±14^{a}	97±35^{b}	32±0^{a}	10±5^{a}	5±3^{a}	11±2^{a}	53±27^{ab}
Luteolin-O-glucoside (isomer 2)	7±2^{a}	7±4^{a}	40±35^{a}	32±8^{a}	102±16^{b}	2±1^{a}	5±2^{a}	n.d	4±0^{a}	20±3^{a}
Luteolin-O-glucoside (isomer 3)	14±6^{ab}	8±1^{ab}	23±10^{b}	8±6^{ab}	21±3^{b}	11±2^{ab}	4±1^{a}	n.d	9±1^{ab}	20±10^{b}
Maslinic acid	488±69^{ab}	1041±121^{bc}	984±499^{abc}	710±153^{ab}	1385±349^{c}	680±184^{ab}	351±101^{a}	497±104^{ab}	364±65^{a}	772±162^{abc}
Metoxypinoresinol glucoside	137±13^{bc}	114±1^{abc}	135±7^{bc}	89±17^{a}	133±28^{bc}	130±17^{abc}	106±13^{ab}	150±8^{c}	119±20^{abc}	108±4^{abc}
Neonuzhenide/oleuropein glucoside (is 1)	144±53^{ab}	239±121^{ab}	161±39^{ab}	122±29^{ab}	210±76^{ab}	208±61^{ab}	375±205^{b}	42±5^{a}	230±81^{ab}	208±84^{ab}
Neonuzhenide/oleuropein glucoside (is 2)	228±42^{cd}	231±24^{cd}	93±44^{a}	114±21^{ab}	310±70^{de}	357±21^{e}	179±16^{abc}	356±29^{e}	202±23^{bc}	167±16^{abc}
Neonuzhenide/oleuropein glucoside (is 4)	48±10^{a}	99±32^{bcd}	88±25^{abcd}	97±25^{bcd}	69±9^{abc}	53±5^{ab}	128±16^{d}	107±4^{cd}	40±5^{a}	56±8^{ab}
Oleanolic acid	2438±437^{ab}	2790±349^{ab}	3775±1693^{b}	2709±1003^{ab}	1586±674^{a}	2713±684^{ab}	1064±298^{a}	1913±280^{ab}	1015±102^{a}	2542±561^{ab}
Oleoside/secologanoside (isomer 1)	203±17^{a}	402±34^{d}	186±37^{a}	278±15^{abc}	310±28^{bcd}	377±28^{cd}	277±52^{abc}	231±14^{ab}	254±59^{ab}	260±36^{ab}
Oleoside/secologanoside (isomer 2)	851±57^{ab}	1566±100^{cd}	855±97^{ab}	1121±85^{b}	1702±168^{d}	1193±138^{bc}	1027±203^{ab}	623±29^{a}	1211±294^{bc}	1153±66^{bc}
Oleuropein	14793±2356^{a}	34335±2802^{e}	21077±1819^{abc}	17190±2044^{ab}	24208±1113^{bcd}	26303±1532^{cde}	17590±5557^{ab}	30795±2207^{de}	19813±4649^{abc}	18180±1040^{abc}
Oleuropein aglycone (isomer 1)	27±8^{a}	125±36^{a}	109±88^{a}	56±19^{a}	107±27^{a}	101±12^{a}	68±27^{a}	67±14^{a}	16±6^{a}	36±17^{a}
Oleuropein aglycone (isomer 2)	26±5^{a}	161±41^{abc}	89±77^{ab}	91±9^{ab}	172±54^{bc}	145±34^{ab}	286±108^{c}	153±5^{abc}	26±16^{a}	32±9^{ab}
Oleuroside	351±21^{ab}	884±23^{f}	308±57^{ab}	528±17^{cd}	714±83^{e}	430±11^{bc}	410±78^{bc}	900±48^{f}	266±61^{a}	583±23^{de}
Phenylethyl primeveroside	49±7^{d}	48±4^{cd}	27±11^{ab}	42±3^{bcd}	27±7^{ab}	58±8^{d}	23±3^{a}	54±7^{d}	30±4^{abc}	17±2^{a}
Quercetin-O-glucoside (isomer 1)	37±8^{ab}	27±2^{ab}	45±27^{ab}	22±9^{a}	142±31^{c}	63±6^{b}	41±5^{ab}	22±2^{a}	22±1^{a}	31±8^{ab}
Quercetin-O-glucoside (isomer 2)	35±3^{a}	17±5^{a}	31±24^{a}	26±3^{a}	96±56^{b}	16±5^{a}	8±3^{a}	9±5^{a}	16±3^{a}	16±6^{a}
Quercetin-O-glucoside (isomer 3)	39±6^{a}	18±4^{a}	40±16^{a}	32±3^{a}	102±37^{b}	30±4^{a}	30±9^{a}	10±4^{a}	22±4^{a}	30±7^{a}
Quinic acid	1792±175^{ab}	2710±112^{b}	2300±831^{ab}	2463±471^{ab}	2095±380^{ab}	1490±103^{a}	1761±89^{ab}	1844±206^{ab}	1469±174^{a}	1862±266^{ab}
Rutin	91±34^{ab}	88±9^{ab}	111±68^{ab}	34±7^{a}	157±33^{b}	99±19^{ab}	54±6^{a}	33±6^{a}	47±5^{a}	34±8^{a}
Taxifolin	186±13^{cde}	151±16^{bcd}	199±12^{de}	31±8^{a}	120±26^{bc}	228±42^{ef}	285±45^{f}	145±11^{bcd}	118±19^{bc}	115±17^{b}
Unknown 1	34±5^{a}	85±35^{ab}	41±35^{a}	157±44^{bc}	101±64^{ab}	251±32^{c}	107±19^{ab}	111±22^{ab}	42±15^{a}	60±47^{ab}
Unknown 2	260±3^{a}	194±20^{a}	202±18^{a}	257±13^{a}	261±61^{a}	240±28^{a}	188±32^{a}	253±16^{a}	221±6^{a}	249±16^{a}
Unknown 4	n.d	43±15^{b}	184±14^{c}	n.d	n.d	n.d	176±37^{c}	n.d	14±0^{a}	n.d
Unknown 5	149±31^{ab}	139±18^{ab}	191±119^{b}	104±33^{ab}	112±23^{ab}	120±43^{ab}	106±36^{ab}	62±9^{ab}	56±11^{a}	151±26^{ab}
Verbascoside	319±89^{a}	1196±205^{ab}	1118±547^{ab}	499±146^{a}	883±429^{a}	521±165^{a}	1941±739^{b}	234±58^{a}	661±189^{a}	508±79^{a}

Lower case letters indicate Tukey's post hoc test differences ($p<0.05$) among different cultivars. Means with a common letter are not significantly different ($p>0.05$); n.d: not detected

Table S2. Quantitative results obtained by LC-IT MS. Data expressed as mg kg^{-1} of olive tissue (DW) ± standard deviation *(cont.)*.

c) Roots	*Arbequina*	*Empeltre*	*Frantoio*	*Hojiblanca*	*Jabali*	*Koroneiki*	*Leccino*	*Mastoidis*	*Menya*	*Picual*
11-Hydroxyiridodial glucoside pentaacetate	123±50^{ab}	278±31^{cd}	77±23^{ab}	151±13^{abc}	131±56^{abc}	345±19^{d}	188±113^{bc}	16±3^{a}	156±66^{abc}	82±42^{ab}
7-deoxyloganic acid	48±12^{a}	16±11^{a}	17±15^{a}	48±19^{a}	81±43^{a}	986±688^{c}	330±126^{b}	58±29^{a}	20±12^{a}	66±41^{a}
Acetoxipinoresinol	155±57^{b}	62±14^{ab}	149±46^{b}	154±78^{b}	147±49^{b}	57±22^{ab}	17±4^{a}	59±9^{ab}	141±22^{b}	51±12^{ab}
Acetoxypinoresinol glucoside	798±156^{bc}	455±116^{ab}	819±76^{bc}	670±95^{abc}	1001±415^{c}	618±32^{abc}	335±26^{a}	657±10^{abc}	803±130^{bc}	262±87^{a}
Betulinic acid	275±24^{c}	269±40^{bc}	244±27^{bc}	183±13^{abc}	112±13^{a}	174±67^{ab}	179±45^{abc}	179±9^{abc}	276±36^{c}	93±18^{a}
Cycloolivil glucoside (isomer 1)	421±79^{cd}	450±99^{d}	164±15^{a}	164±30^{a}	211±76^{ab}	411±44^{cd}	378±90^{bcd}	370±33^{bcd}	263±60^{abcd}	251±100^{abc}
Cycloolivil glucoside (isomer 2)	190±77^{ab}	426±38^{c}	354±97^{bc}	255±119^{abc}	117±45^{a}	104±7^{a}	142±27^{a}	258±59^{abc}	232±58^{abc}	210±71^{ab}
Elenolic acid glucoside (isomer 1)	286±91^{abc}	767±68^{d}	289±98^{abc}	172±25^{ab}	292±79^{abc}	442±75^{bc}	547±256^{cd}	420±32^{abc}	335±82^{abc}	128±37^{a}
Elenolic acid glucoside (isomer 2)	121±47^{abc}	284±31^{d}	83±22^{ab}	29±8^{a}	76±26^{a}	302±43^{d}	204±98^{bcd}	225±35^{cd}	118±30^{abc}	27±11^{a}
Hydroxy oleuropein	152±36^{a}	450±311^{a}	246±133^{a}	197±90^{a}	184±124^{a}	248±102^{a}	193±28^{a}	429±20^{a}	372±126^{a}	86±8^{a}
Hydroxypinoresinol glucoside	528±48^{bc}	472±32^{abc}	713±27^{de}	545±27^{bc}	347±124^{a}	738±49^{de}	818±52^{e}	603±24^{cd}	573±56^{bcd}	426±66^{ab}
Ligstroside	1502±234	4061±222	1778±268	877±155	1961±1026	3526±739	3111±1616	2542±180	2446±729	914±325
Lucidumoside C	270±89^{ab}	1045±823^{b}	426±259^{ab}	273±146^{ab}	313±277^{ab}	582±307^{ab}	438±92^{ab}	868±88^{ab}	688±255^{ab}	113±12^{a}
Maslinic acid	3082±777^{ab}	3000±774^{ab}	2181±436^{a}	2514±106^{ab}	2535±465^{ab}	2824±692^{ab}	3351±601^{ab}	2780±842^{ab}	3897±391^{b}	2497±170^{ab}
Metoxypinoresinol glucoside	87±6^{bc}	60±12^{ab}	101±15^{c}	97±13^{c}	72±22^{abc}	36±1^{a}	44±10^{a}	83±4^{bc}	45±3^{a}	89±20^{bc}
Neonuzhenide/oleuropein glucoside (isomer 1)	101±47^{abc}	195±52^{abc}	70±19^{ab}	26±9^{a}	101±49^{abc}	339±85^{c}	269±230^{bc}	113±13^{abc}	92±17^{ab}	34±13^{ab}
Neonuzhenide/oleuropein glucoside (isomer 2)	90±35^{ab}	156±12^{bc}	70±30^{ab}	31±9^{a}	138±59^{ab}	394±57^{d}	170±55^{bc}	260±36^{c}	117±19^{ab}	42±9^{a}
Neonuzhenide/oleuropein glucoside (isomer 4)	15±2^{a}	35±1^{cd}	16±2^{ab}	7±1^{a}	13±4^{a}	39±5^{d}	26±5^{bc}	34±5^{cd}	16±3^{ab}	8±3^{a}
Oleanolic acid	53±11^{a}	43±11^{a}	45±4^{a}	38±4^{a}	30±6^{a}	43±10^{a}	49±15^{a}	38±6^{a}	36±5^{a}	34±4^{a}
Oleoside/secologanoside (isomer 1)	69±15^{ab}	204±15^{e}	61±12^{a}	64±13^{a}	70±13^{abc}	147±26^{de}	130±50^{cd}	125±7^{bcd}	57±9^{a}	38±5^{a}
Oleoside/secologanoside (isomer 2)	267±44^{abc}	323±60^{bc}	244±24^{abc}	366±99^{c}	388±98^{c}	164±37^{ab}	300±24^{abc}	165±7^{ab}	139±29^{a}	227±70^{abc}
Oleuropein	17093±6267^{ab}	37381±5024^{d}	13510±1354^{ab}	6736±898^{a}	11165±4890^{a}	33302±2467^{cd}	24029±8228^{bc}	33049±1922^{cd}	13790±4379^{ab}	6545±2577^{a}
Quinic acid	252±14^{a}	295±50^{a}	356±239^{a}	383±65^{a}	377±73^{a}	245±85^{a}	288±36^{a}	239±5^{a}	370±125^{a}	311±87^{a}
Unknown 1	133±63^{ab}	266±77^{bcd}	124±68^{ab}	81±23^{a}	203±89^{abc}	423±84^{d}	241±68^{abc}	341±11^{cd}	94±31^{ab}	66±36^{a}
Unknown 2	141±25^{ab}	162±17^{b}	135±7^{ab}	123±20^{ab}	103±26^{a}	103±16^{a}	117±9^{ab}	142±14^{ab}	144±3^{ab}	116±29^{ab}
Unknown 3	57±38^{ab}	185±21^{c}	33±14^{a}	18±2^{a}	38±8^{ab}	131±31^{bc}	108±55^{abc}	185±63^{c}	40±10^{ab}	16±8^{a}
Verbascoside	6055±1827^{a}	2778±715^{a}	5655±643^{a}	9359±5461^{a}	8883±4473^{a}	8887±2296^{a}	9913±1546^{a}	4799±1477^{a}	4990±783^{a}	8695±3120^{a}
Isoverbascoside	693±89^{ab}	366±79^{a}	1185±72^{ab}	1237±716^{ab}	1150±306^{ab}	711±19^{ab}	924±68^{ab}	760±52^{ab}	724±157^{ab}	1442±681^{b}

Lower case letters indicate Tukey's post hoc test differences (p<0.05) among different cultivars. Means with a common letter are not significantly different (p>0.05)

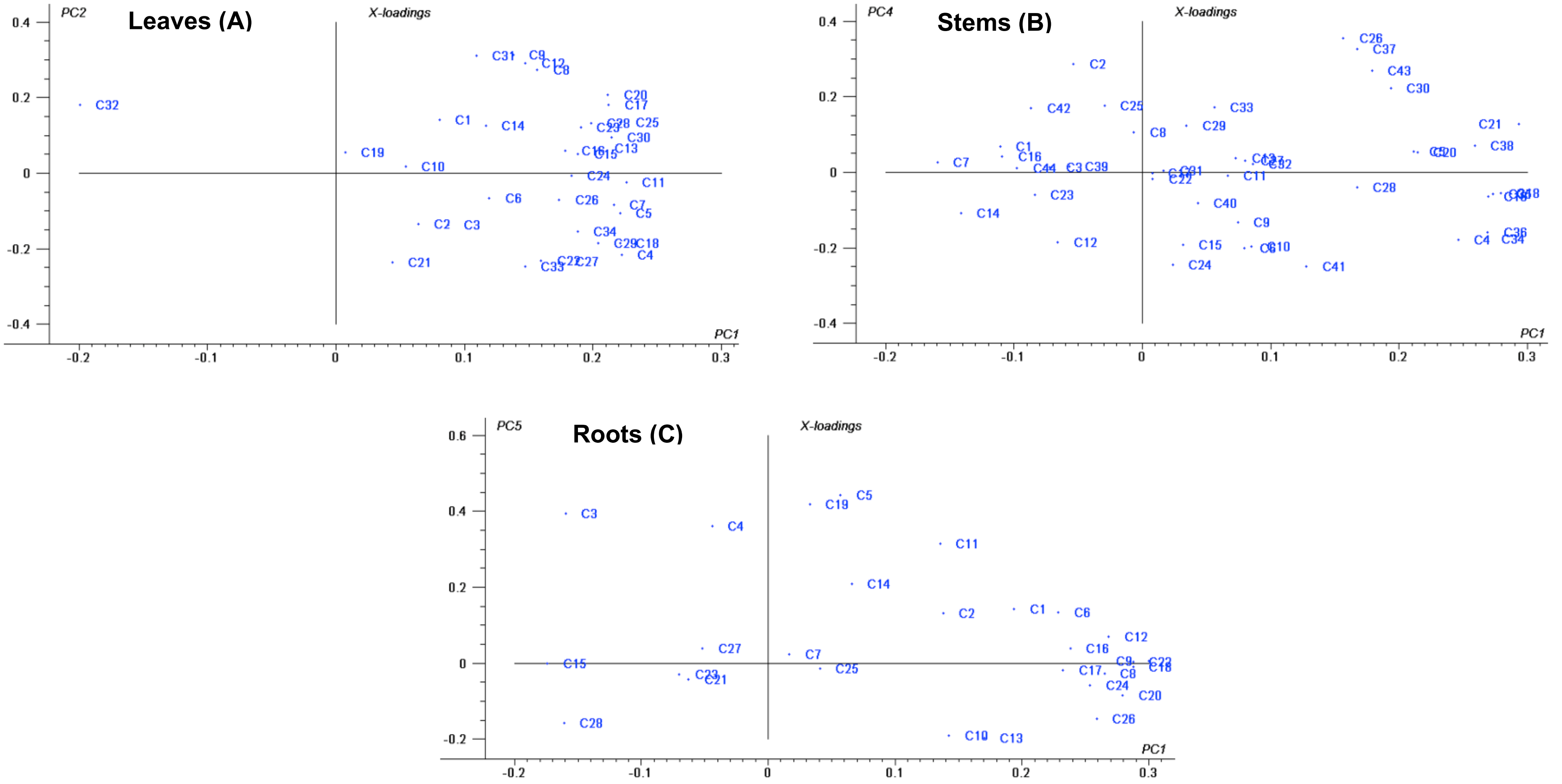

Figure S1. Principal component analysis (PCA) loadings plots representing the two principal components (PCs) providing the best samples' grouping for each olive tissue: leaves (A), stems (B) and roots (C). *Compound numbers at the end of the document.

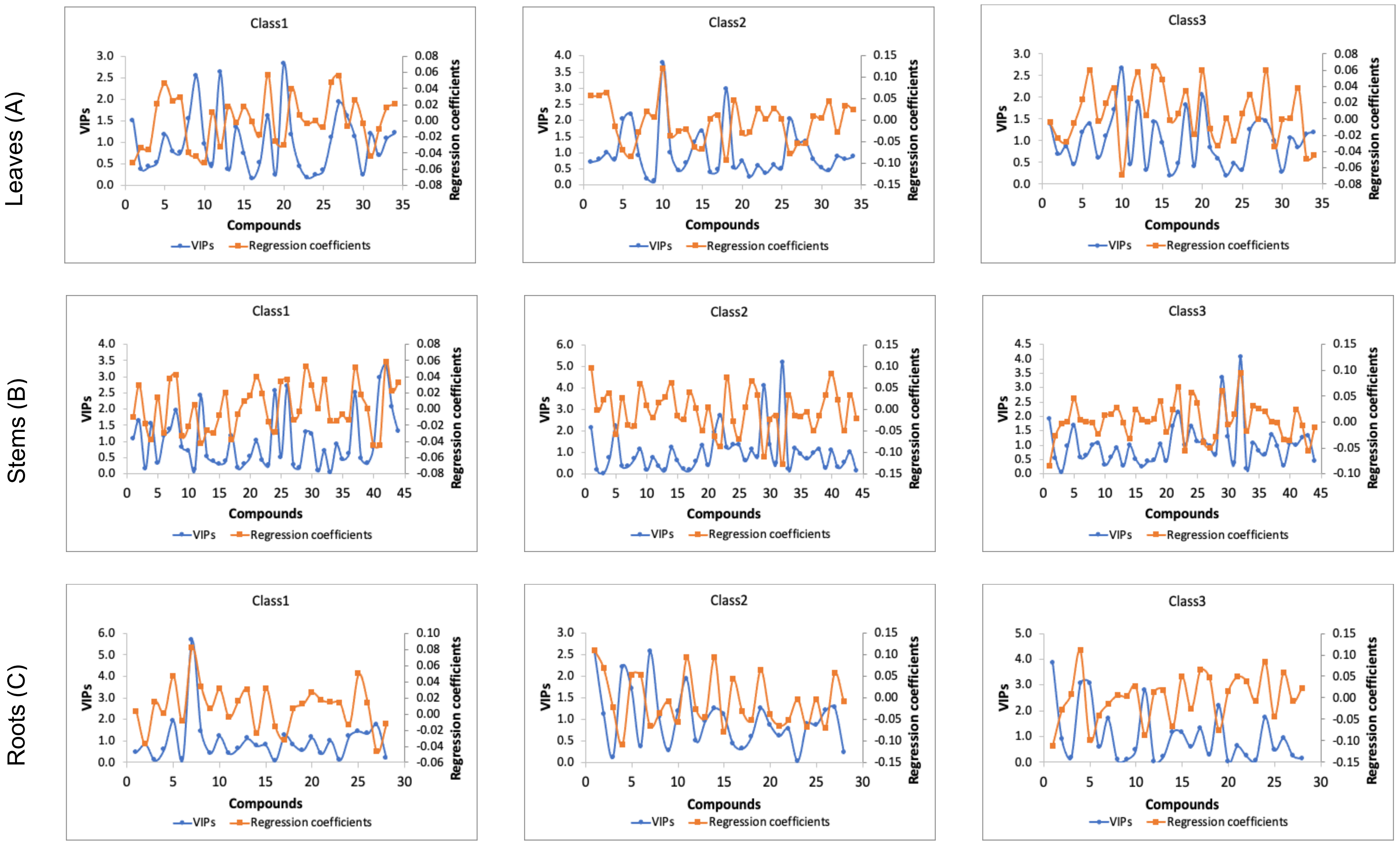

Figure S2. Representation of the regression coefficients and variable importance on the projection (VIP) values of each metabolite quantified in the three olive tissues under study: leaves (A), stems (B) and roots (C). Class 1: high resistant cultivars; class 2: resistant, moderately susceptible, and susceptible cultivars; class 3: extremely susceptible cultivars. *Compound numbers at the end of the document.

***Compound numbers in Figure S1 and S2.**

LEAVES		STEMS		ROOTS	
**Code**	**Compound name (leaves)**	**Code**	**Compound name**	**Code**	**Compound name**
C1	Aldehydic form of decarboxymethyl elenolic acid glucoside	C1	11-Hydroxyiridodial glucoside pentaacetate	C1	11-Hydroxyiridodial glucoside pentaacetate
C2	Apigenin 7-*O*-glucoside	C2	Acetoxipinoresinol	C2	7-deoxyloganic acid
C3	Apigenin *O*-rutinoside	C3	Acetoxypinoresinol glucoside	C3	Acetoxipinoresinol
C4	Betulinic acid	C4	Aldehydic form of decarboxymethyl elenolic acid glucoside	C4	Acetoxypinoresinol glucoside
C5	Chrysoeriol O-glucoside	C5	Betulinic acid	C5	Betulinic acid
C6	Demethyl oleuropein	C6	Cyanidin *O*-glucoside	C6	Cycloolivil glucoside (isomer 1)
C7	Diosmin	C7	Cycloolivil	C7	Cycloolivil glucoside (isomer 2)
C8	Elenolic acid glucoside (isomer 1)	C8	Demethyl oleuropein	C8	Elenolic acid glucoside (isomer 1)
C9	Elenolic acid glucoside (isomer 2)	C9	Dihydrokaempferol	C9	Elenolic acid glucoside (isomer 2)
C10	Gallocatechin	C10	Dihydroquercetin 3-O-glucoside	C10	Hydroxy oleuropein
C11	Hydroxytyrosol	C11	Elenolic acid glucoside (isomer 1)	C11	Hydroxypinoresinol glucoside
C12	Hydroxytyrosol glucoside	C12	Elenolic acid glucoside (isomer 2)	C12	Ligstroside
C13	Ligstroside	C13	Hydroxy oleuropein	C13	Lucidumoside C
C14	Lucidumoside C	C14	Hydroxypinoresinol glucoside	C14	Maslinic acid
C15	Luteolin 7-*O*-glucoside (isomer 1)	C15	Hydroxytyrosol glucoside	C15	Metoxypinoresinol glucoside
C16	Luteolin *O*-glucoside (isomer 2)	C16	Ligstroside	C16	Neonuzhenide/oleuropein glucoside (isomer 1)
C17	Luteolin *O*-glucoside (isomer 3)	C17	Lucidumoside C	C17	Neonuzhenide/oleuropein glucoside (isomer 2)
C18	Maslinic acid	C18	Luteolin 7-*O*-glucoside (isomer 1)	C18	Neonuzhenide/oleuropein glucoside (isomer 4)
C19	Neonuzhenide/oleuropein glucoside (isomer 1)	C19	Luteolin *O*-glucoside (isomer 2)	C19	Oleanolic acid
C20	Neonuzhenide/oleuropein glucoside (isomer 3)	C20	Luteolin *O*-glucoside (isomer 3)	C20	Oleoside
C21	Neonuzhenide/oleuropein glucoside (isomer 4)	C21	Maslinic acid	C21	Secologanoside
C22	Oleanolic acid	C22	Metoxypinoresinol glucoside	C22	Oleuropein
C23	Oleoside	C23	Neonuzhenide/oleuropein glucoside (isomer 1)	C23	Quinic acid
C24	Secologanoside	C24	Neonuzhenide/oleuropein glucoside (isomer 2)	C24	Unknown 1
C25	Oleuropein	C25	Neonuzhenide/oleuropein glucoside (isomer 4)	C25	Unknown 2
C26	Oleuropein aglycone (isomer 1)	C26	Oleanolic acid	C26	Unknown 3
C27	Oleuropein aglycone (isomer 2)	C27	Oleoside	C27	Verbascoside
C28	Oleuroside	C28	Secologanoside	C28	Isoverbascoside
C29	Quinic acid	C29	Oleuropein		
C30	Rutin	C30	Oleuropein aglycone (isomer 1)		
C31	Unknown 1	C31	Oleuropein aglycone (isomer 2)		
C32	Unknown 2	C32	Oleuroside		
C33	Unknown 5	C33	Phenylethyl primeveroside		
C34	Verbascoside	C34	Quercetin *O*-glucoside (isomer 1)		
		C35	Quercetin *O*-glucoside (isomer 2)		
		C36	Quercetin *O*-glucoside (isomer 3)		
		C37	Quinic acid		
		C38	Rutin		
		C39	Taxifolin		
		C40	Unknown 1		
		C41	Unknown 2		
		C42	Unknown 4		
		C43	Unknown 5		
		C44	Verbascoside		
